# Engineered zinc-finger transcription factors activate *OCT4* (*POU5F1*), *SOX2*, *KLF4*, *c-MYC* (*MYC*) and miR302/367

**DOI:** 10.1093/nar/gku243

**Published:** 2014-05-03

**Authors:** Qingzhou Ji, Ashley L. Fischer, Clyde R. Brown, Erik R. Eastlund, Tamar Dvash, Bonan Zhong, Mark A. Gerber, Ian Lyons, Scott W. Knight, Carol A. Kreader

**Affiliations:** Sigma-Aldrich Corporation, Saint Louis, MO 63103, USA

## Abstract

Artificial transcription factors are powerful tools for regulating gene expression. Here we report results with engineered zinc-finger transcription factors (ZF-TFs) targeting four protein-coding genes, *OCT4*, *SOX2*, *KLF4* and *c-MYC*, and one noncoding ribonucleic acid (RNA) gene, the microRNA (miRNA) miR302/367 cluster. We designed over 300 ZF-TFs whose targets lie within 1 kb of the transcriptional start sites (TSSs), screened them for increased messenger RNA or miRNA levels in transfected cells, and identified potent ZF-TF activators for each gene. Furthermore, we demonstrate that selected ZF-TFs function with alternative activation domains and in multiple cell lines. For *OCT4*, we expanded the target range to −2.5 kb and +500 bp relative to the TSS and identified additional active ZF-TFs, including three highly active ZF-TFs targeting distal enhancer, proximal enhancer and downstream from the proximal promoter. Chromatin immunoprecipitation (FLAG-ChIP) results indicate that several inactive ZF-TFs targeting within the same regulatory region bind as well as the most active ZF-TFs, suggesting that efficient binding within one of these regulatory regions may be necessary but not sufficient for activation. These results further our understanding of ZF-TF design principles and corroborate the use of ZF-TFs targeting enhancers and downstream from the TSS for transcriptional activation.

## INTRODUCTION

Various classes of naturally occurring deoxyribonucleic acid (DNA)-binding molecules, including zinc-finger (ZF) proteins (1,2), triplex forming oligos (3), meganucleases (4) and transcription activator-like effectors (TALEs) (5–7), as well as the CRISPR *cas9* protein-guide ribonucleic acid (RNA) system (8,9), have been engineered to bind sequence-specific endogenous target sites. Such sequence-specific binding proteins, when coupled to various functional domains, are powerful tools for modifying the genome and regulating gene expression. ZF proteins have been highly characterized for such uses (1,2). These modular proteins consist of multiple ZF domains, each recognizing three base pairs of DNA linked together to generate a protein that binds specific DNA sequences. For the most part, ZF and other DNA-binding proteins have been fused with the endonuclease domain from FokI to allow site-specific cutting, followed by insertion and/or deletion (10,11). More recently, these proteins have been fused to transcriptional activation or repression domains, such as VP16 from the herpes simplex virus or a Krueppel-associated box (KRAB) domain. These engineered transcription factors (TFs) effectively up or downregulate target gene expression when delivered into cells (12–18).

Engineered ZF- and TALE-TFs have been applied to upregulate a number of genes encoding TFs, including genes typically overexpressed for cellular reprogramming (6,17,19,20). For example, ZF-TFs were previously reported to increase *OCT4* expression in embryonic stem (ES) cells (17), and ZFPs fused to a transcriptional repressor domain have been shown to upregulate *OCT4* (21). TALE-TFs have been successfully designed to activate *SOX2* and *KLF4* (6). TALE-TFs were initially reported to activate *OCT4* only when used in combination with global epigenetic inhibitors (19). Recently, however, stably expressed TALE-TFs were shown to upregulate *OCT4* and successfully replace exogenous OCT4 to reprogram mouse embryonic fibroblasts (22).

One of the challenges in designing ZF- and TALE-TFs is determining the best locations to target on the gene of interest. Chromatin accessibility, proximity to the promoter and the ability to bind, recruit accessory factors and not block sites on the chromatin where other factors must bind may all be important. Previous reports have targeted DNase-hypersensitive regions (16,23) or, more specifically, the proximal promoter and/or a known enhancer (6,20,22). Few ZF- or TALE-TFs that target outside these known gene elements have been tested.

To investigate the rules for designing potent engineered ZF-TFs more comprehensively, we performed a systematic screen of ZF-TFs designed to upregulate endogenous gene expression. We designed and screened over 300 promoter region-targeting ZF constructs fused with the p65 subunit of NF-ĸB, and from these we identified ZF-TFs that activate *OCT4*, *SOX2*, *KLF4* and *c-MYC*. From these initial results, we expanded our evaluation to screen and identify active ZF-TFs that target the upstream enhancer region of *OCT4*, as well as several that upregulate a noncoding RNA transcript. In addition, we demonstrate that selected ZF-TFs function with alternative activation domains and in multiple cell lines.

## MATERIALS AND METHODS

All reagents are from Sigma-Aldrich, except where noted otherwise.

### ZF-TFs design and construction

Five- or six-fingered ZF proteins targeting human *OCT4* (*POU5F1*), *SOX2*, *KLF4*, c-*MYC* (*MYC*) and the miR302/367 transcription unit were designed using the Sigma-Aldrich CompoZr^®^ ZFN algorithm. ZF-TFs with the NF-ĸB p65 activation domain were constructed by CompoZr ZFN Operations in pcDNA3.1 (Invitrogen). The three most active *OCT4*-, *SOX2-*, *KLF4-* and *MYC*-p65 constructs are available from Sigma-Aldrich as CZFA17184, CZFA2136, CZFA1504 and CZFA1041, respectively. ZF-2xp65, ZF-VP16 and ZF-VP64 constructs were generated by ligating ZF fragments into *Acc*65I-*Bam*H1-digested vectors containing the respective activation domain with all other elements identical to the p65 constructs (i.e. same promoter, polyA sequence, etc.).

### Cell culture and transfection

The human cell lines HEK293, K562 and BJ fibroblasts were obtained from the American Type Culture Collection. HEK293 and BJ cells were grown in Dulbecco's modified Eagle's medium, supplemented with 10% FBS, 2-mM l-glutamine, 1-mM sodium pyruvate and 0.1-mM nonessential amino acids; K562 cells were grown in Iscove's Modified Dulbecco's Medium, supplemented with 10% FBS (fetal bovine serum) and 2-mM L-glutamine. Cultures were split 1 day before transfection and were at ∼70% confluency for HEK293 and BJ cells, and ∼0.5 million cells per milliliter for K562 at the time of transfection. For the initial screen of ZF-TFs targeting −800 to +200 on OCT4, SOX2, KLF4 and MYC, HEK293 cells were nucleofected with Amaxa® Cell Line 96-well Nucleofector® Kit SF (Lonza) and program CM-130 or Nucleofector Solution V (Lonza) and program Q-001. For subsequent experiments, HEK293 cells were transfected with Lipofectamine 2000 (Life Technologies) or TransIT-LT1 (MirusBio) in 12-well cultures according to the manufacturer's instructions. BJ cells were transfected with TransIT-2020 (Mirus Bio) and K562 cells were nucleofected with Nucleofector Solution V and program T-016 following the manufacturer's protocols.

### Real-time RT-PCR analysis

**Figure 1.**
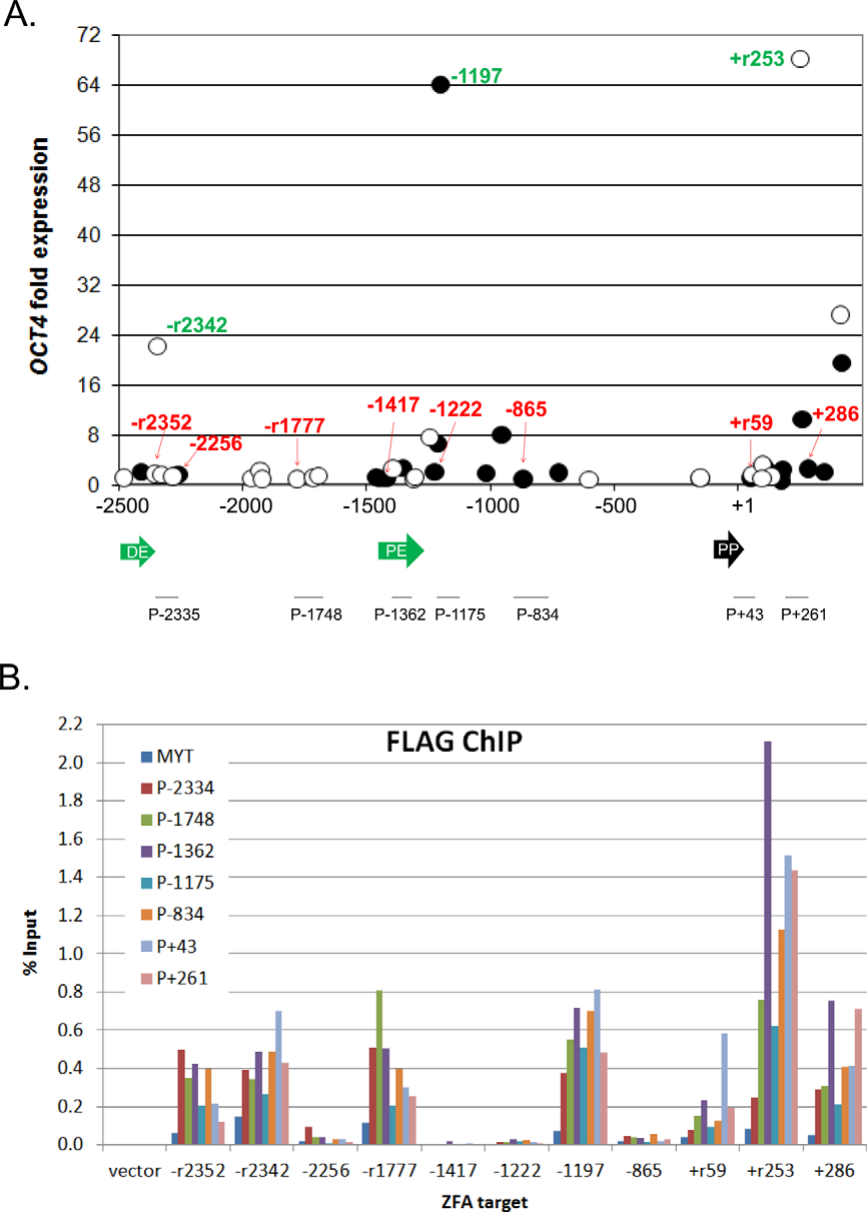
Results for engineered ZF-TFs targeting endogenous *OCT4* in HEK293 cells. (**A**) Real-time quantitative reverse transcriptase polymerase chain reaction (qRT-PCR) results for *OCT4* mRNA levels. Expression plasmids encoding ZF-TFs with the NF-ĸB p65 activation domain were transfected into HEK293 cells. Control cells were transfected with vectors encoding GFP (Green Fluorescent Protein). After 48 h, the cells were harvested and total RNA was isolated. The levels of *OCT4* mRNA and *PPIA* (cyclophilin A) endogenous control mRNA were measured by qRT-PCR. The *OCT4*/*PPIA* ratio was used for normalizing *OCT4* expression. Values plotted are the fold *OCT4* expression compared to the GFP control. ZF-TFs that bind on the forward strand (closed circles) and reverse strand (open circles) were graphed according to their target location between −2500 bp and +500 bp of the major *OCT4* TSS. Those used for FLAG-ChIP-qPCR are indicated with their position in green for active ZF-TFs and red for inactive ZF-TFs. Positions of the distal enhancer (DE), proximal enhancer (PE) and proximal promoter (PP), also known as conserved regions 4, 2 and 1, respectively (25,40), are indicated below the x-axis. Positions of ChIP-qPCR amplicons are also indicated below the x-axis and are named according to the midpoint of the amplicon. (**B**) FLAG-ChIP-qPCR results. Chromatin immunoprecipitation was performed with cross-linked chromatin from HEK293 cells transfected with each of the 11 ZF-TF constructs indicated in (A) and anti-FLAG M2 antibody, as described in the Materials and Methods section. qPCR was performed with a series of primers across the ZF-TF target region on *OCT4* [indicated below the x-axis in (A)], as well as primers that amplify a closed chromatin region on MYT where the ZF-TFs should not bind. Percent of input was calculated and plotted for each. Percent input was ≤0.003% for all negative control, mouse IgG ChIPs (not shown) and for vector alone with anti-FLAG.

**Figure 2.**
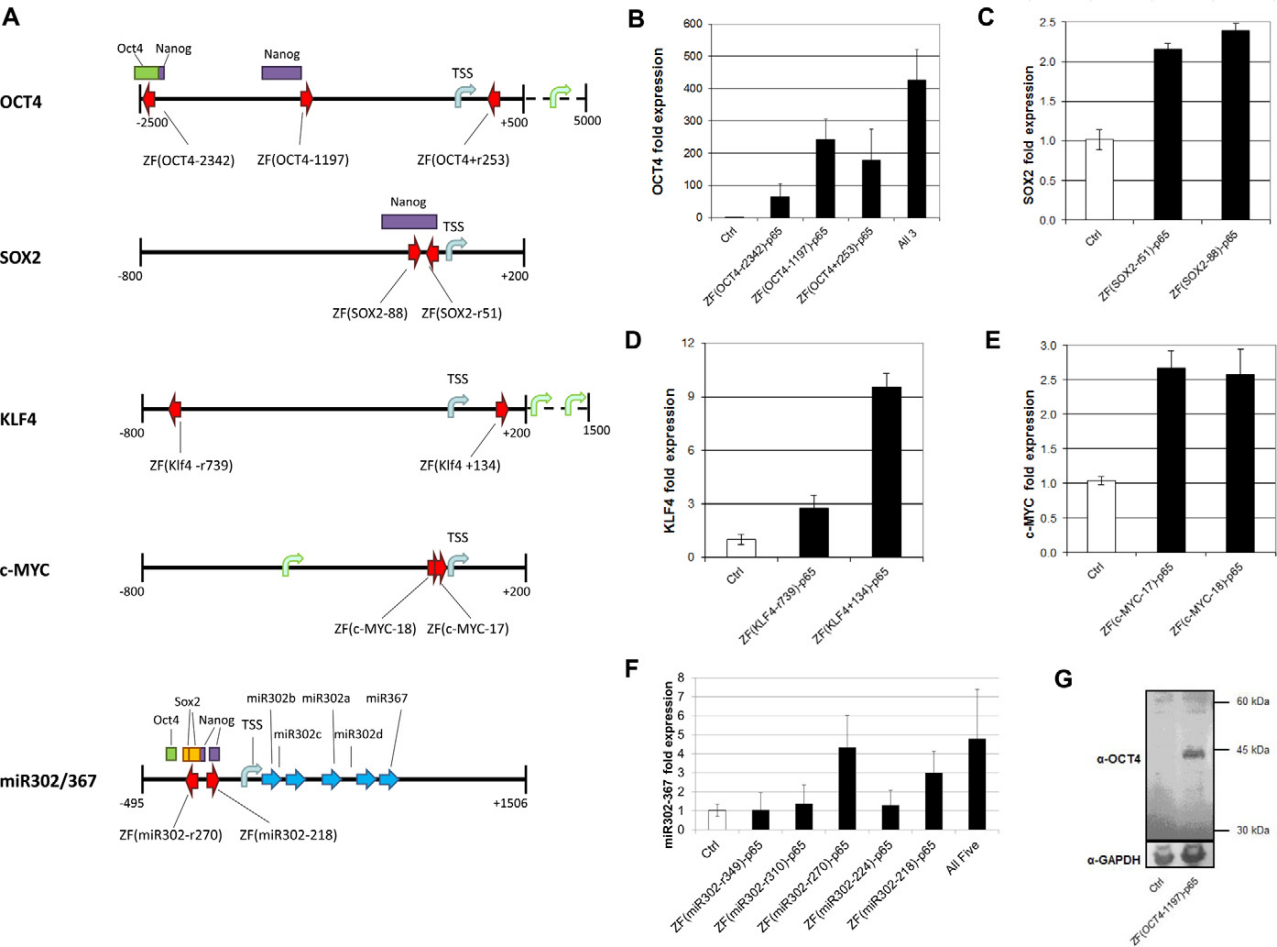
Targeted gene activation by ZF-TFs in HEK293 cells. (**A**) Schematic representation of each targeted gene and the predicted ZF-TF-binding sites. Arrows pointing to the right indicate ZF-TFs that bind the forward strands; arrows pointing to the left indicate ZF-TFs that bind the reverse strand. Binding sites for endogenous NANOG, POU5F1 and SOX2 in H1-hESCs, as reported in the UCSC Preview Genome Browser [Human Feb. 2009 (GRCh37/hg19) Assembly, http://genome-preview.ucsc.edu/, Transcription Factor ChIP-seq from ENCODE track], are indicated by purple, green and yellow bars, respectively. Locations for *miR302a/b/c/d* and *miR367*, as well as OCT4, SOX2 and NANOG-binding sites within the miR302/367 target region are shown (29). Transcriptional activation by representative ZF-TFs for *OCT4* (**B**), *SOX2* (**C**), *KLF4* (**D**), *c-MYC* (**E**) and miR302/367 (**F**) in HEK293 cells is shown. Binding sites are indicated for only the two most active ZF-TFs on miR302/367. Averages from three biological replicates are plotted with error bars representing one standard deviation. (**G**) ZF(*OCT4–*1197)-p65 upregulates OCT4 protein levels in HEK293 cells. Total cell lysates were prepared from HEK293 cells transfected with either GFP control plasmid or plasmid containing the ZF(*OCT4–*1197)-p65 expression cassette. Western analysis was conducted using an OCT4-specific antibody and anti-GAPDH as described in the Materials and Methods section.

**Figure 3.**
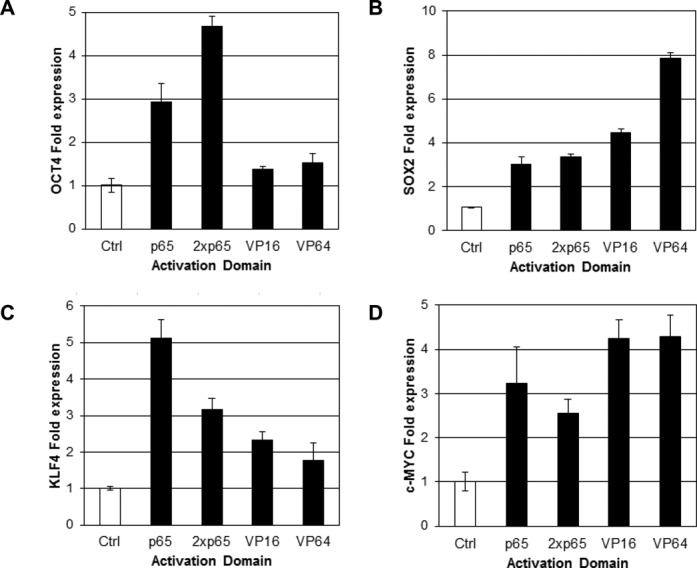
ZF-TF activity varies with different activation domains. p65, 2xp65, VP16 or VP64 activation domains were fused to the following ZF-TFs: ZF(*OCT4*+181), ZF(*SOX2–*88), ZF(*KLF4*+134) or ZF*(c-MYC*-17), and transcriptional activation of *OCT4* (**A**), *SOX2* (**B**), *KLF4* (**C**) and *c-MYC* (**D**) by these constructs in HEK293 cells is shown. Averages from three biological replicates are plotted with error bars representing one standard deviation.

**Figure 4.**
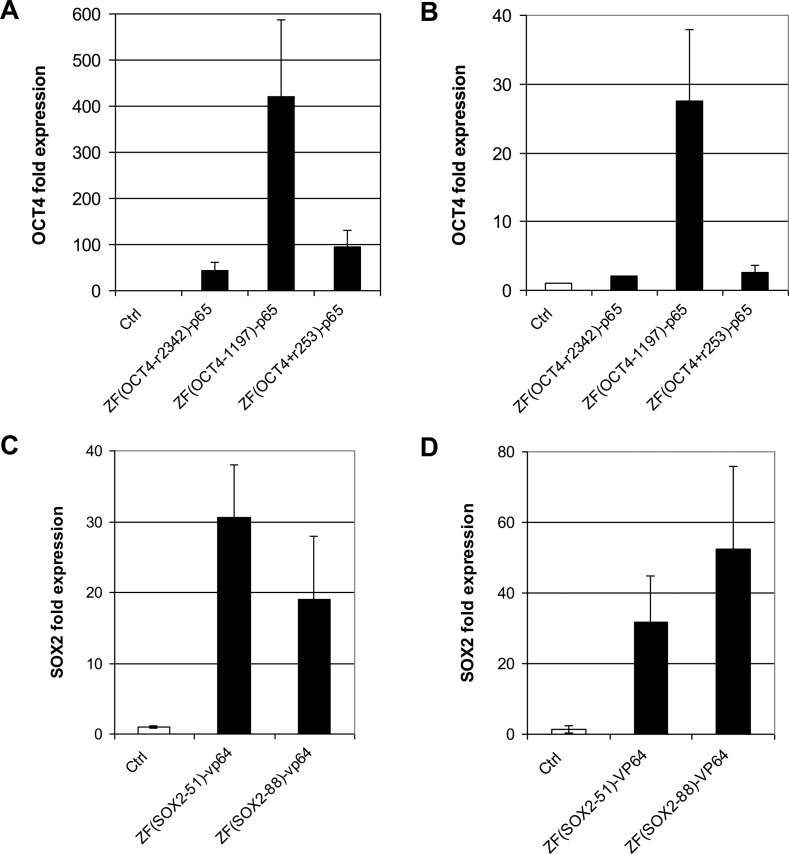
ZF-TFs activated targeted genes in both K562 and BJ cells. ZF-TFs targeting the indicated genes were tested in K562 cells (**A**) and (**C**) or BJ fibroblasts (**B**) and (**D**). Averages from three biological replicates are plotted with error bars representing one standard deviation.

For messenger RNA (mRNA) results shown in Figures 1–4, cells were harvested 48 h post-transfection. Total RNA was isolated from all cells using RNeasy Plus 96 Kit (Qiagen) or RNeasy Plus Mini Kit (Qiagen). The levels of target gene mRNA and *PPIA* (cyclophilin A) endogenous control mRNA were measured by real-time quantitative reverse transcriptase polymerase chain reaction (qRT-PCR) using Quantitative RT-PCR ReadyMix (QR0100), as recommended by the manufacturer. Human *OCT4*, *SOX2* and *KLF4* *and c-MYC* Taqman gene expression assays (Hs03005111_g1, Hs04234836_s1, Hs00358836_m1 and Hs00153408_m1, respectively) and Human *PPIA* Endogenous Control Taqman assay (4326316E) were from Life Technologies. All results were normalized to *PPIA* using the delta *C_t_*/*C_q_* method.

For microRNA (miRNA) results shown in Figure 2, cells were harvested 72 h post-transfection, and total RNA was isolated using Total RNA Purification Kit (Norgen Biotek Corp.). Human *miR-302a* and Human SNORD48 (small nucleolar RNA, C/DR box 48) Taqman miRNA assays were from Life Technologies. *miR-302a* and SNORD48 endogenous control RNA levels were measured by reverse transcription and qPCR with M-MLV (Moloney Murine Leukemia Virus) Reverse Transcriptase and JumpStart Taq ReadyMix for Quantitative PCR from Sigma-Aldrich Biotechnology following Life Technologies’ thermocycling conditions for miRNA RT and Sigma's for qPCR. *miR-302a* levels were normalized to SNORD48 using the delta *C_q_* method.

### Western analysis

Total cell lysates were prepared from cells at 48 h (Supplementary Figure S2A) or 72 h (Figure 1G and Supplementary Figure S3) post-transfection. After washing cells in phosphate buffered saline, lysates were made directly in Laemmli buffer and incubated in boiling water for 5 min. Total protein was resolved by sodium dodecyl sulphate-polyacrylamide gel electrophoresis (12% Tris-Glycine), followed by transfer to PVDF (Polyvinylidene fluoride) membranes for immunoblotting. Blots were probed with anti-OCT4 (Abcam), anti-GAPDH (Glyceraldehyde 3-phosphate dehydrogenase) or anti-FLAG M2 (F1804), followed by peroxidase-conjugated secondary antibodies and development with TMB reagent (3,3',5,5'-Tetramethylbenzidine; T8665).

### Chromatin immunoprecipitation

Eleven ZF-TF constructs were subcloned from their original CompoZr vector, which contained a single C-terminal FLAG tag, into pVAX-NELD-GFP (CompoZr GFP-ZFN expression vector) to generate N-terminal triple FLAG-tagged constructs. HEK293 cells were seeded at 10^6^ cells/well in 6-well plates, transfected with 4 μg ZF-TF construct using Lipofectamine 2000, and incubated 48 h, as described above. In preliminary experiments, half the amount of ZF-TF construct was tested and incubation was reduced to 24 h; however, results were essentially the same, so the conditions above were used for all results presented. Chromatin immunoprecipitation (ChIP) was performed using Sigma's Imprint® Chromatin Immunoprecipitation Kit (CHP1) as instructed in the User Guide, with the following modifications. Preliminary experiments showed that Protein A Magnetic Sepharose Xtra (GE28-9670-56) with anti-mouse IgG (Immunoglobulin G) bridging antibody (M7023) gave higher signal and lower background than the strip wells provided with CHP1, so the magnetic Sepharose was used for results presented here. Preliminary experiments also showed no difference in FLAG-ChIP results from ZF-TF-transfected cells with formaldehyde cross-linking for 5 versus 10 min, nor with shearing for 2, 3 or 4 × 10 min, 30 s on/30 s off at maximum energy on a Diagenode Biorupter. Analysis on an Agilent Bioanalyzer High Sensitivity chip showed DNA was sheared to generate fragments between ∼350 and 50 bp in all cases, with peaks between 200 and 120 bp. Therefore, 8 min of formaldehyde cross-linking and 3 × 10 min shearing were used for the results presented. Each sheared chromatin preparation was split into three equal amounts, and ChIP was performed with anti-FLAG (F1804), anti-H3K27me3 (SAB4800015) or mouse IgG (I5381). qPCR primers were designed with PrimerBLAST (Supplementary Table S6). qPCR was performed with KiCqstart PCR mix (KCQS01) and primers at 0.4 μM on a Stratagene MX3000P using the following cycling parameters: denature at 94^○^C, 3 min followed by 40 cycles of 94^○^C, 15 s and 60^○^C, 1 min.

## RESULTS

### Identifying active ZF-TFs

To identify artificial TFs that upregulate targeted gene expression, we initially designed ZF-TFs to an ∼1-kb region of the promoter for *OCT4*, *SOX2*, *KLF4* and *c-MYC*. Designs spanned a region from −800 bp to +200 bp relative to the major transcriptional start site (TSS). In an effort to conduct an unbiased screen that would identify potent transcriptional activators, we designed ZF-TFs predicted to bind throughout the 1-kb promoter region, as evenly spaced as possible. Note that CompoZr ZF design rules preclude design of ZFs targeting repetitive regions or other sequences sufficiently similar to allow off-target binding. As a consequence, target sites could not be evenly distributed. Most of the ZF domains consisted of six ZF subunits with a few five-finger constructs, resulting in ZF-TFs with either an 18- or 15-bp DNA-recognition site (Supplementary Tables S1–S5). Each six or five-finger ZF coding sequence assembly was cloned between an N-terminal nuclear localization signal and a C-terminal NF-ĸB p65 activation domain to generate a series of ZF-TF expression constructs for each gene. Each series included 59–120 ZF-TFs that target either the forward or the reverse DNA strand.

Engineered ZF-TFs were screened in HEK293 cells for the ability to upregulate target gene expression. Plasmid DNA encoding each ZF-TF was transfected into cells and mRNA levels from each target gene were measured using qRT-PCR ∼48 h after transfection. Potent ZF-TF activators were identified for each target gene. Five ZF-TFs targeting the *OCT4* −800 to +200 promoter region resulted in 2-fold or higher *OCT4* transcript levels compared to control cells (Figure 1A and Supplementary Table S1). Similarly, seven ZF-TF designed to *SOX2*, eight activators for *KLF4* and five activators for *c-MYC* gave at least a 2-fold increase in corresponding transcript levels (Supplementary Figure S1A–D and Supplementary Tables S2–S5). Results with representative ZF-TFs that increased target gene transcript levels greater than 2-fold are shown in Figure 2. It should be noted that although basal levels of *OCT4, SOX2*, *KLF4* and *c-MYC* transcripts are low in HEK293 cells without ZF-TFs, all four transcripts were detected within the range of their Taqman assays (*C_q_* = 30–32). Furthermore, all positive results were verified by performing three biological replicate experiments (i.e. seeding and transfecting from three separate starter cultures on three different days).

Regulation of gene expression is often controlled by *cis-*acting sequences that can be located at great genetic distances form the core promoter. The *OCT4* proximal and distal enhancers have been previously defined as key regions important in regulating *OCT4* expression in different cell types (24,25,40). ZF-TFs and TALE-TFs that target the enhancer regions of *SOX2* and *OCT4*, respectively, have been shown to activate gene expression (20,22). However, none of the previous studies comprehensively probed the intervening sequences for effective target sites. To determine if ZF-TFs targeting enhancer and intervening regions outside the core promoter would also be capable of upregulating *OCT4* expression, we designed and tested an additional 27 ZF-TFs that target between −2500 and −800 relative to the TSS. Ten of the 27 ZF-TFs targeting this region resulted in increases in *OCT4* mRNA levels of more than 2-fold (Figure 1 and Supplementary Table S1). Both ZF(*OCT4–*1197)-p65, which targets the *OCT4* proximal enhancer, and ZF(*OCT4*−r2342)-p65, which targets the *OCT4* distal enhancer, increased *OCT4* transcript levels more than 16-fold in HEK293 cells. Therefore, both enhancers are good targets for artificial transcription activators.

Encouraged by our ability to identify active ZF-TFs designed to bind upstream of the core promoter, we also designed and tested six ZF-TFs that target the region downstream from the core promoter, from +200 bp to +500 bp relative to the *OCT4* TSS. All six ZF-TFs increased *OCT4* mRNA levels more than 2-fold in HEK293 cells, and three ZF-TFs, ZF (*OCT4*+r253)-p65, ZF(*OCT4*+414)-p65 and ZF(*OCT4*+r420)-p65 activated *OCT4* transcript levels more than 16-fold (Figure 1 and Supplementary Table S1).

To verify that increased activation of *OCT4* expression stimulated by ZF-TFs resulted in functional mRNA, we transfected HEK293 cells with either control plasmid or plasmid carrying the most potent *OCT4* ZF-TF, ZF(*OCT4–*1197)-p65. Three days post-transfection, cells were harvested and OCT4 protein levels were determined by western analysis. OCT4 was readily detected in total cell lysates when cells were transfected with the ZF-TF, compared to undetectable levels of OCT4 in control cells (Figure 2G). These results indicate that transcriptional activation of *OCT4* by ZF-TFs produces functional mRNA.

Since the three most active ZF-TFs for *OCT4* target three separate regulatory elements (Figure 1A), we tested whether the three together would upregulate *OCT4* expression to higher levels than each individually. As shown in Figure 2B, the amount of *OCT4* transcript detected in HEK293 cells transfected with all three ZF-TFs was nearly equal to the sum of that in cells transfected with single ZF-TFs. Note that the total amount of DNA transfected was the same for three ZF-TF constructs (0.53 μg each) as for one ZF-TF (1.6 μg). Therefore, multiple ZF-TFs can work in concert, as shown by others (16,23,26).

To determine what role the ability of ZF-TFs to bind their targets plays in *OCT4* activation, we performed FLAG-ChIP-qPCR. Eleven constructs were selected for this analysis, two with target sites closest to each of the three most active ZF-TFs and two targeting the intervening regions. All constructs had N-terminal triple FLAG tags. HEK293 cells were transfected with each of these 11 constructs as well as the vector, the latter of which expresses FLAG and p65, but no ZF. Anti-FLAG western blots show that all 11 FLAG-tagged constructs were expressed, although one (ZF(*OCT4–*1777)-p65) appears mostly degraded. After 48 h, cells were cross-linked, and chromatin was prepared and sheared. Each batch of sheared chromatin was split into three equal portions, and ChIP was performed with anti-FLAG, anti-H3K27me3 (positive control) or mouse IgG (negative control). qPCR was performed on ChIP DNA with primers targeting ≤65 bp from each ZF-TF target site, as well as primers to a region of H3K27-methylated, closed chromatin in the distal promoter of *MYT* (Myelin transcription factor) (27), where the ZF-TFs should not bind. Anti-H3K27me3 selectively pulled down *MYT* (i.e. at levels greater than non-target regions on *OCT4*) from all 12 chromatin preps (Supplementary Figure S2B), demonstrating that all were ChIP-suitable. IgG pulled down no or barely detected amounts of DNA (≤0.003% of input) in all cases. FLAG-ChIP for cells transfected with vector alone also gave no or barely detected amounts of DNA (≤0.003% of input) for all primers. On the other hand, anti-FLAG ChIP with chromatin from cells expressing each of the 11 ZF-TFs pulled down more DNA from *OCT4* upstream regions than from non-target *MYT* (Figure 1B), although none showed much if any enrichment at their specific target site. The latter is likely due to the scanning mechanism used by ZF proteins to find their targets and because ZF-TFs are expressed in excess over their target sites, as discussed below. Only three of the eight ZF-TFs tested that do not or only weakly upregulate *OCT4* (ZF(*OCT4*−r2352, −1777 and +286)-p65) gave FLAG-ChIP yields similar to the three most active ZF-TFs (ZF(*OCT4*−r2342, −1197 and +r253)-p65). Two of these three inactive/weakly active ZF-TFs bind within known regulatory regions (ZF(*OCT4*−r2352)-p65, distal enhancer, inactive; ZF(*OCT4*+286)-p65, proximal promoter, weakly active), while the third binds in the region between the proximal and distal enhancers (ZF(*OCT4–*1777)-p65, inactive). The entire ChIP experiment was repeated with a second set of cultured and transfected HEK293 cells, and similar results were obtained. Therefore, as expected, not all ZF-TFs that bind *OCT4* can upregulate *OCT4*, but unexpectedly, not all ZF-TFs that appear to bind efficiently in known enhancer or promoter regions can upregulate *OCT4*.

To test whether ZF-TFs could also be used to activate noncoding genes, as recently shown for TALE-TFs (23), we designed 60 ZF-TFs targeting miR302/367 from −0.5 kb to +1.5 kb relative to the TSS and screened the artificial activators in HEK293 cells for the ability to increase mature miR-302a levels. The miR302/367 cluster, like most miRNA genes, is structured and regulated similar to protein coding genes. The location of its Pol II promoter, TSS and several TF-binding sites have been reported by others (28,29). Ten ZF-TFs identified from the screen increased endogenous miR-302a levels more than 2-fold when introduced into HEK293 cells (Supplementary Table S5). Two ZF-TFs led to a greater than 4-fold increase in miR-302a (Figure 2F). Co-transfection of the five ZF-TFs that target known TF-binding sites into the same cells did not result in higher levels of miR-302a than the two most active ZF-TFs did individually (Figure 2F).

### ZF-TF activity varies with different activation domains

The activation domain linked to a ZF-TF can have a large influence on the level of transcriptional activation achieved by such artificial TFs (16,23,30). Using ZF-TFs we identified in the initial screen described above, we further optimized targeted gene activation by replacing the NF-ĸB p65 domain in four of the ZF constructs with three alternative activation domains. ZF-TFs targeting *OCT4*, *SOX2*, *KLF4* and *c-MYC* were tested with VP16, VP64 or 2Xp65 (two tandem copies of the p65 domain) in addition to p65. For *KLF4*, the original ZF-TF containing the p65 activation domain induced the greatest increase in transcript levels (Figure 3C). On the other hand, activation of *OCT4*, *SOX2* and *c-MYC* was increased by expressing respective ZF-TFs with one of the alternative activation domains. Transcript levels for *SOX2* were highest in cells expressing the ZF-TF with a VP64 domain (Figure 3B), and for *c-MYC*, the ZF-TFs with VP16 and VP64 gave slightly higher transcript levels than did p65 (Figure 3D). These results are consistent with previous reports that demonstrated an ∼5-fold activation of *SOX2* activation with an artificial TALE-VP64 domain (6). For consistency, we used a ZF-TF targeting *OCT4* within its core promoter region to evaluate the effects of different activation domains on *OCT4* expression. *OCT4* mRNA levels were highest when the selected ZF-TF contained the 2xp65 domain, with little *OCT4* activation observed in cells transfected with ZF-TFs containing the VP16 or VP64 domains (Figure 3A). Western blot analysis showed no correlation between ZF-TF expression and activity. Since all constructs had only a single FLAG tag, some were not detected and most were barely detected above background on the western (Supplementary Figure S3). For example, only the p65 version of ZF(OCT4+181) was detected by anti-FLAG western, however, the 2xp65 construct was the best activator for *OCT4*. Similarly, the VP16 and VP64 versions of ZF(KLF4+134) gave the strongest western bands, but the p65 construct gave the best activation of *KLF4*.

### Engineered ZF-TFs are active in multiple cell types

All of the ZF-TFs described here were identified based on their ability to activate targeted gene transcription in HEK293 cells. As different cell types can express different TFs, and the chromatin state at promoters can vary depending on the cell type, we were interested in whether active ZF-TFs identified in HEK293 cells would also activate target gene expression in other cell types. We tested selected ZF-TFs in K562 human chronic myelogenous leukemia cells and BJ human foreskin fibroblast cells. BJ fibroblasts were of particular interest, as these cells are often used for cellular reprogramming by overexpressing the same genes targeted by our ZF-TFs (31). As shown in Figure 4A and B, all three ZF-TFs targeting *OCT4* that were tested [ZF(*OCT4*−r2342)-p65, ZF(*OCT4–*1197)-p65 and ZF(*OCT4*+r253)-p65] increased *OCT4* mRNA levels in both K562 and BJ fibroblasts, although the relative increase in *OCT4* transcript levels in BJ fibroblasts (Figure 4B) was much less than that observed in HEK293 (Figure 2B) and K562 (Figure 4A) cells. Strikingly, ZF(*OCT4–*1197)-p65 induced *OCT4* mRNA levels several 100-fold in K562 cells. *SOX2*-ZF-TFs were also tested in K562 and BJ cells (Figure 4C and D, respectively). Both ZF(*SOX2*-r51)-VP64 and ZF(*SOX2–*88)-VP64 induced an increase in *SOX2* mRNA levels relative to the control that was substantially greater than that observed in HEK293 cells (Figure 4C and D versus Figure 2C). It should be noted, however, that whereas the native, non-induced *OCT4* mRNA levels were similar and within the range of the qRT-PCR assay in the three cell lines tested (Cq ∼32–33), non-induced *SOX2* mRNA levels were not. The basal level of *SOX2* mRNA was considerably higher in HEK293 (Cq ∼29), and too low for reliable detection in BJ and K562 (Cq ∼38). Therefore, the fold-expression values for *SOX2* in BJ and K562 are approximations only. Regardless of these differences, the results demonstrate that active ZF-TFs are capable of upregulating targeted genes in multiple cell types and have access to the ZF recognition sequence in at least the three cell types tested.

## DISCUSSION

Previous research on ZF- and TALE-TFs has primarily focused on characterizing a limited number of artificial TFs, often targeted to DNase-hypersensitive or known TF-binding regions (6,12–17,19). To further investigate the rules for designing potent engineered ZF-TFs, we conducted a systematic screen of several hundred ZF-TFs with target sites spread as evenly as possible from −800 to +200 relative to TSSs and identified several that activate each of *OCT4*, *SOX2*, *KLF4* and *c-MYC*. Our success rate was lower than reported in other studies, as expected since we did not confine our ZF-TF target sites to known regulatory elements. The active ZF-TFs identified upregulated their target genes from 2-fold to over 500-fold, depending on the cell type, activation domain fused to the ZF-TF and the gene targeted. The levels of activation achieved with several ZF-TFs were similar to or higher than those previously reported for ZF-TFs targeting *OCT4* (17,22) and TALEs targeting *SOX2* and *KLF4* (6). In addition, the upregulation of *OCT4* with ZF-TFs described here did not require additional globally active epigenetic agents nor stable expression from an integrated construct, as was reported previously for TALE-TFs targeting *OCT4* (19,22).

To further explore locations within a gene that might be good sites for ZF-TFs to bind and upregulate transcription, we designed ZF-TFs targeting the upstream enhancer as well as the region downstream from the TSS for *OCT4*. Strikingly, all six ZF-TFs designed to the downstream region were able to activate endogenous *OCT4* in HEK293 cells, and three of six designs were capable of activating *OCT4* more than 16-fold in HEK293 cells (Figure 1). Several active ZF-TFs for *SOX2*, *KLF4* or *c-MYC* also bind downstream of the primary TSS (Supplementary Figure S1). Likewise, a target region 1 kb downstream from the TSS was previously reported for active engineered ZF-TFs (16). The ability of ZF-TFs that bind downstream of the TSS to activate gene expression suggests that these ZF-TFs may lead to transcription initiation from an alternative TSS or that the ZF-TF may recruit the initiation complex, followed by displacement of the ZF-TF during transcription. Indeed, alternative TSSs have been annotated nearly 4 kb downstream from the major *OCT*4 TSS, and 0.7 and 1.3 kb downstream from the major *KLF*4 TSS (32,33). Since the qPCR assays used for *OCT*4 and *KLF*4 amplify regions common to all their transcripts, it is unknown which transcript or transcripts are upregulated by the ZF-TFs targeting downstream from the major TSS. On the other hand, neither SOX2 nor MYC has a known alternative downstream TSS, and none of their downstream targeting ZF-TFs gave >2-fold activation (Supplementary Figure 1A and C and Supplementary Tables S2 and S4).

Perhaps not surprisingly, two of the three best activators for *OCT4* were located in the upstream enhancer regions of the gene, confirming the results of others (6,20,22) that artificial TF designs should not be limited to the region of just a few hundred base pairs around a TSS. ZF-TFs may act as ‘pioneer TFs’, which bind the enhancer regions of repressed genes to recruit other factors, open up the chromatin and activate transcription (34), as has been suggested for the reprogramming factors *OCT4*, *SOX2*, *KLF4* and *c-MYC* (OSKM) themselves (35). Binding sites for TFs such as NANOG, OCT4 and p300 are found in the *OCT4* enhancer region (Figure 2A) (33). Introduction of ZF(*OCT4–*1197)-p65, which is expected to bind in this region, upregulates OCT4 mRNA and protein, consistent with the pioneer TF model.

The promoter region of the miR302/367 cluster also contains binding sites for OCT4 and NANOG, as well as SOX2 (Figure 2A), which have been shown to regulate miR302/367 expression (28,29). The two most active ZF-TFs for upregulating the miR302/367 cluster, ZF(*miR302*−r270)-p65 and ZF(*miR302–*218)-p65, have predicted DNA-binding sites that overlap these SOX2 and NANOG-binding sites. In addition, two of the best ZF-TF activators for *SOX2* target the region adjacent to a NANOG-binding site. These results suggest that selecting ZF-TFs that bind near or overlap with sites used by naturally occurring TFs, especially ‘pioneer TF’-binding sites, may be a successful strategy for designing active ZF-TFs.

During the process of screening for active ZF-TFs, we identified multiple active ZF-TFs within defined areas of each gene tested, indicating that some areas are prime locations for functional ZF-TFs. In contrast, there are also regions where multiple ZF-TFs were predicted to bind, but failed to activate transcription. One explanation for these results may be that inactive ZF-TFs simply do not bind the DNA and, therefore, fail to recruit the cellular factors necessary to induce transcription. Alternatively, the epigenetic status at a promoter, including DNA methylation patterns and chromatin structure, influences the ability of TFs to gain access to the DNA and likely also plays a role in whether a particular artificial TF will bind the DNA and lead to transcriptional activation. Our most active ZF-TFs target within regions reported to be DNAse hypersensitive, and therefore accessible, in human H1 and H7 ES cells (33). For *SOX2, KLF4* and *c-MYC*, the same regions were shown to be DNase hypersensitive in several somatic cell lines as well (Supplementary Figure S4B–D). However, for *OCT4* they were not (Supplementary Figure S4A). Although HEK293 cells, which were used for most of the results presented here, were not among the cells evaluated, HEK293T and K562 cells were. None of our active ZF-TF target sites for *OCT4* are within DNase-hypersensitive regions reported for HEK293T or K562. These results support those of Perez-Pinera *et al.* (26), which show active TALE-TFs targeting regions on *IL1RN*, *KLK3* and *CEACAM5* that are not DNase hypersensitive. Therefore, a good strategy for artificial activator design may be to target regions that are DNase hypersensitive in cells where the gene of interest is highly expressed rather than to target DNase-hypersensitive regions in the cells used for gene activation where expression is low or undetectable, i.e. location may be more important than DNase accessibility.

Intriguingly, for all the gene targets evaluated, we found the binding sites for ZF-TFs that did increase levels of expression overlapped or were in very close proximity to those of ZF-TFs that did not. These results again may be due to active ZF-TFs binding their targets and inactive ZF-TFs not effectively binding, or alternatively, the position or orientation of the activation domain relative to the TSS, other *cis*-elements, or *trans-*factors required to initiate transcription may be critical for ZF-TF-induced gene activation. Shifting the binding site a few base pairs may present the activation domain to other factors in an orientation that succeeds or fails to initiate transcription.

Our ChIP results for 11 of the ZF-TFs targeting *OCT4* include examples of ZF-TFs that do not bind efficiently, those that appear to bind efficiently but do not activate, as well as three that bind and activate *OCT4* expression >20-fold (Figure 1). Two of the three inactive or weakly active ZF-TFs that appear to bind efficiently target within or close to known regulatory features, ZF(*OCT4*−r2352)-p65, which binds within the distal enhancer and ZF(*OCT4* +286)-p65, which targets downstream from the proximal promoter. Weakly active ZF-TF, ZF(*OCT4*+286)-p65, targets a site 33 bp further from the proximal promoter than does the active ZF-TF, ZF(*OCT4*+r253)-p65. However, ZF-TFs ZF(*OCT4*+r414 and +440)-p65, which were not included in the ChIP analysis, target sites even further downstream and both activate *OCT4* expression ∼20-fold. On the other hand, inactive ZF-TF, ZF(*OCT4*−r2352)-p65, targets a site 10 bases further into the distal enhancer than does the active ZF-TF, ZF(*OCT4* –r2342)-p65. Since the most active ZF-TFs for *OCT4* target sites slightly downstream from known regulatory elements, the distal enhancer, proximal enhancer and proximal promoter (Figure 1A), it is possible that ZF-TF binding within these elements can interfere with their function.

Although our ChIP results did not show much if any enrichment of *OCT4* ZF-TFs at their specific target sites, all 11 tested were more enriched across *OCT4* than on closed chromatin at MYT (Figure 1B). Our ZF proteins are derived from Zif268 (also known as Egr-1) (36), which has been shown to search for its target sequences by a scanning process, whereby it binds nonspecific DNA sites and slides with intersegment transfer until it finds its specific target (37). Since our ZF-TFs were expressed from a cytomegalovirus promoter, their levels were likely much higher than most endogenous TFs. Furthermore, each ZF-TF has fewer specific target sites (two) than most endogenous TFs (tens to hundreds). As a consequence, ZF-TF levels would be in large excess over their specific target sites, and cross-linking to nonspecific sites would be expected. Our results are consistent with those of Falahi et al., who showed enrichment of the E2C ZFP ∼100 and 700 bp upstream from its target site on *Her2/neu* (*ERBB2*) (38). Others who have reported ChIP results for highly expressed, engineered ZF-TFs or TALE-TFs have reported more selective enrichment (39) or have only presented results at the specific target sites (20–22).

In addition to the target location for the activating ZF-TF, the attached functional domain and the cell type into which the ZF-TF is delivered can influence targeted gene activation levels (Figure 3). The greatest transcriptional activation of *SOX2* in HEK293 cells was observed using a ZF-TF containing a VP64 activation domain. A ZF-TF containing a 2xp65 activation domain was most efficient at upregulating *OCT4* in HEK293 cells, but the same ZF-TF with a VP16 or VP64 domain failed to activate *OCT4* transcription efficiently. These results indicate that the cellular machinery is present in HEK293 cells to activate transcription from VP16/64, but suggest that different factors are required at different promoters or positions within the promoter for optimal transcriptional activation. All ZF-TFs that activated transcription in one cell type also led to increased mRNA levels in the other cell types tested; however, differences in relative activation were observed in some cases. As previously noted, differences in the epigenetic state of a promoter or levels of endogenous accessory factors in different cell types likely contribute to the ability of a ZF-TF to activate transcription in different cell types.

Several artificial TFs have been designed to activate genes involved in reprogramming, but there is yet to be a report of using such tools alone to reprogram cells. However, with the additional ZF-TFs reported here, the number of activators targeting such genes has substantially increased. Having a collection of these tools may allow for further analysis of the transcriptional requirements for cellular reprogramming. Other groups have reported that combinations of TALE-TFs can act synergistically to upregulate targeted genes, including miR302/367 (23,26). Although we did not detect any additional increase in mature miR-302a when up to five ZF-TFs targeting this gene were used (Figure 2F), we did see an additive increase in OCT4 mRNA levels with our three most active ZF-TFs to *OCT4* (Figure 2B). It will be interesting to see if some combination of ZF-TFs can activate expression to the levels needed for reprogramming. Based on these results, it is intriguing to speculate that artificial TFs may one day be used as a novel tool for generating induced pluripotent stem cells.

## SUPPLEMENTARY DATA

Supplementary Data are available at NAR Online.

SUPPLEMENTARY DATA
