# Supplementary material for: Engineered zinc-finger transcription factors activate OCT4 (POU5F1), SOX2, KLF4, c-MYC (MYC) and miR302/367
Source: Nucleic Acids Res. 2014 May 3;42(10):6158–67. doi: 10.1093/nar/gku243 (PMC4041418; doi:10.1093/nar/gku243)
Supplement: SUPPLEMENTARY DATA [file supp_42_10_6158__index.html]

Engineered zinc-finger transcription factors activate OCT4 (POU5F1), SOX2, KLF4, c-MYC (MYC) and miR302/367 — Engineered zinc-finger transcription factors activate OCT4 (POU5F1), SOX2, KLF4, c-MYC (MYC) and miR302/367 — SUPPLEMENTARY DATA 

# Engineered zinc-finger transcription factors activate *OCT4* (*POU5F1*), *SOX2*, *KLF4*, *c-MYC* (*MYC*) and miR302/367

## SUPPLEMENTARY DATA

**Files in this Data Supplement:**

- SUPPLEMENTARY DATA
