# Supplementary material for: Engineered zinc-finger transcription factors activate OCT4 (POU5F1), SOX2, KLF4, c-MYC (MYC) and miR302/367
Source: Nucleic Acids Res. 2014 May 3;42(10):6158–67. doi: 10.1093/nar/gku243 (PMC4041418; doi:10.1093/nar/gku243)
Supplement: SUPPLEMENTARY DATA [file supp_gku243_nar-01672-h-2013-File003.pdf]

Supplementary Table 1. ZF-TFs for OCT4

Name	target site	Fingers	Oct4 activation (Fold)
ZF(OCT4-2404)	atTGAGATGGCTGGGGAGGGgcctcctc	6	2.0
ZF(OCT4-2256)	gaCCTGTGGCAGGTATTGAAatgcacgc	6	1.6
ZF(OCT4-1459)	tgTGAGGGGATTGGGACTGGggggttgg	6	1.2
ZF(OCT4-1444)	acTGGGGGGTTGGGGAGCAGgaagcagt	6	1.0
ZF(OCT4-1417)	tcCCCAGGGGAGCCATCCAGgcccattc	6	1.0
ZF(OCT4-1351)	ctGGGGACCGGGATTGTCCAgccaaggc	6	2.6
ZF(OCT4-1222)	ggCCTAGGGCTGGAGGCCTGggccaggg	6	2.0
ZF(OCT4-1210)	gaGGCCTGGGCCAGGGAGGTgggggagg	6	6.6
**ZF(OCT4-1197)**	**ggGAGGTGGGGGAGGGAGAAcggggcct**	**6**	**64.0**
ZF(OCT4-1015)	ttGAAGGGGAAGTAGGGGCCaacccctt	6	1.9
ZF(OCT4-952)	tgGAAGACGGAGGGGTGGGGggatgggg	6	7.9
ZF(OCT4-865)	atTTGGCAGGCTGGGCAGATggtgccag	6	1.0
ZF(OCT4-720)	atGCATTGAGGGATaGCGCCAcnnnnnn	6	1.9
ZF(OCT4+52)	tcCCCATGGCGGGACACCTGgcttcgga	6	1.1
ZF(OCT4+104)	tgGTGGAGGTGATGGGCCAGgggggccg	6	3.0
ZF(OCT4+111)	ggTGATGGGCCAGGGGGGCCggagccgg	6	2.5
ZF(OCT4+123)	ggGGGGCCGGAGCCGGGCTGggttgatc	6	1.6
ZF(OCT4+130)	cgGAGCCGGGCTGGGTTGATcctcggac	6	2.0
ZF(OCT4+176)	ccCTCCTGGAGGGCCAGGAAtcggccg	6	1.7
ZF(OCT4+177)	ccTCCTGGAGGGCCAGGaatcgggccnn	5	0.7
ZF(OCT4+181)	ctGGAGGGcCAGGAATCGGGCcnnnnnn	6	2.5
ZF(OCT4+261)	ttCTGTGGGGGGATGGCGTActgtgggc	6	10.5
ZF(OCT4+286)	ggCCCCAGGTTGGAGTGGGGctagtgcc	6	2.6
ZF(OCT4+349)	aaGCAGGAGTCGGGGTGGAGagcaactc	6	2.1
ZF(OCT4+420)	gtGAAGCTGGAGAAGGAGAAgctggagc	6	19.5
ZF(OCT4-r2475)	tcTCAGAGGAGGGGGAGGGGcaggcctg	6	1.1
ZF(OCT4-r2352)	ngGGGTGGGAGGAACATGCTtcggaaca	6	1.7
**ZF(OCT4-r2342)**	**ggGGCCTGGTGGGGGTGGGAggaacatg**	**6**	**22.1**
ZF(OCT4-r2325)	aaAGGCAGGTAGATTATGGGgcctggtg	6	1.6
ZF(OCT4-r2279)	gcAGTGGGGTTGGAGCTGTGttcacttc	6	1.3
ZF(OCT4-r1960)	tcGAAGGCCAGAAGGCCAGGtctggact	6	1.0
ZF(OCT4-r1926)	caGAGGGGGCTCTGGGCCAGggctcccc	6	2.2
ZF(OCT4-r1917)	tgGGGGCTCCAGAGGGGGCTctgggcca	6	0.8
ZF(OCT4-r1777)	agTATCCTGAGGCTCATGCTgctggtct	6	0.9
ZF(OCT4-r1712)	gtGGAGGGGTGCAGGAAGGCtgccctaa	6	1.1
ZF(OCT4-r1690)	caGAGGAGGTGGCGAGTGATttgtggag	6	1.3
ZF(OCT4-r1391)	ctTGAATGGGCCTGGATGGCtcccctgg	6	2.5
ZF(OCT4-r1307)	ggGGAAGGGGGCAGgACAATGgccttgg	6	0.8
ZF(OCT4-r1301)	nnACTGGGGGAAGGGGGCAGgacaatgg	6	1.1
ZF(OCT4-r1243)	agGGGGTGGGGGGTGTGGAGaaaaaata	6	7.6
ZF(OCT4-r601)	nnTCAGGAACCCAGGTGcttgaccccct	5	0.8
ZF(OCT4-r151)	nnGGGGAAAACCGGGAGacacaactggc	5	1.0
ZF(OCT4+r59)	ccGCCATGGGGAAGGAAGGCgccccaag	6	1.6
ZF(OCT4+r92)	ggGGCGAGAAGGCAaAATCTGaagccag	6	1.5
ZF(OCT4+r99)	ncTGGAGGgGGCGAGAAGGCAaaatctg	6	0.9
ZF(OCT4+r101)	caCCTGGAGGGGGCGAGAAGgcgaaatc	6	3.1
ZF(OCT4+r136)	gcCCGGCTCCGGCCCCCCTGgcccatca	6	1.3
**ZF(OCT4+r253)**	**atACGGCGGGGGGCATGGGGgaatcccc**	**6**	**68.1**
ZF(OCT4+r414)	gcACCAGGGGTGACGGTGCAgggctccg	6	27.2

Supplementary Table 2. ZF-TFs for SOX2

Name	Target Site	Fingers	SOX2 activation (Fold)
ZF (SOX2-387)	ccTTAGCTGCTtCCCGCGtcccatcctc	5	1.4
ZF (SOX2-r414)	ggGGTGGGGCAGGGcACAGTGggtggct	6	1.3
ZF (SOX2-r405)	aaGGTGCGGGGGTGGGGCAGggcacagt	6	1.2
ZF (SOX2-r400)	caGCTAAGGTGCGGGGGTGGggcagggc	6	1.2
ZF (SOX2-r394)	ggGAAGCAGCTAAGGTGCGGgggtgggg	6	1.5
ZF (SOX2-r385)	gaTGGGACGCGGGAaGCAGCTaaggtgc	6	1.2
ZF (SOX2-r379)	agGATGGGACGCGGGAAgcagctaaggt	5	1.6
ZF (SOX2-r379)	atGAGGATGGGACGCGGGAAgcagctaa	6	1.2
ZF (SOX2-378)	taAATGAGgATGGGAcGCGGGAagcagc	6	1.2
ZF (SOX2-273)	ccTTCGAAAAGgCGTGTGgtgtgacctg	5	1.1
ZF (SOX2-270)	tcGAAAAGgCGTGTGgTGTGACctgttg	6	1.3
ZF (SOX2-266)	aaAGGCGTGTGgTGTGACctgttgctgc	5	1.1
ZF (SOX2-260)	gtGTGGTGtGACCTGTTGCTGcgagagg	6	0.9
ZF (SOX2-253)	gtGACCTGTTGCTGCGAGAGgggataca	6	1.3
ZF (SOX2-251)	gaCCTGTTGCTGCGAGAGGGgatacaaa	6	1.1
ZF (SOX2-245)	ttGCTGCGAGAGGGGATacaaaggtttc	5	1.5
ZF (SOX2-218)	ctCAGTGGCTGGCAGGCTGGctctggga	6	1.2
ZF (SOX2-215)	agTGGCTGgCAGGCTgGCTCTGggagcc	6	1.2
ZF (SOX2-211)	gcTGGCAGgCTGGCTCTGGGAgcctcct	6	1.5
ZF (SOX2-r307)	acTAAGACTACGTGGGTtttttttttctt	5	1.2
ZF (SOX2-r298)	agTGGGTAaACAGCAcTAAGACtacgtg	6	1.4
ZF (SOX2-r273)	ccACACGCCTTTTCGAAggaagtgggta	5	1.1
ZF (SOX2-r253)	tcTCGCAGCAACAGGTCacaccacacgc	5	1.3
ZF (SOX2-r252)	ctCTCGCAGCAACAGGTcacaccacacg	5	1.1
ZF (SOX2-146)	ccGCGCGGCCGGCGGCGCGGgaggcccc	6	2.0
ZF (SOX2-140)	ggCCGGCGGCGCGGGAGGCCccgccccc	6	1.0
ZF (SOX2-111)	ttCATGCAaAACCCGGCAGCGaggctgg	6	1.1
ZF (SOX2-r198)	gaGGGGGAGGAGGCTCCCAGagccagcc	6	1.4
ZF (SOX2-r192)	ggCGAGGAGGGGGAGGAGGCtcccagag	6	1.5
ZF (SOX2-r184)	agGGGGCAGGCGAGGAGGGGgaggaggc	6	2.4
ZF (SOX2-r181)	agGAGGGGGCAGGCGAGGAGggggagga	6	2.2
ZF (SOX2-r178)	ggGAGGAGGGGGCAGGCGAGgaggggga	6	2.1
ZF (SOX2-r171)	naGGCCGGgGGAGGAGGGGGCaggcgag	6	1.3
ZF (SOX2-r168)	ggGGAGGCcGGGGGAGGAGGGggcaggc	6	1.3
ZF (SOX2-r155)	cgCCGGCCGCGCGGgGGAGGCcgggggga	6	1.8
ZF (SOX2-r148)	ccGCGCCGCCGGCCGCGCGGgggaggcc	6	1.2
ZF (SOX2-r139)	gcGGGGCCtCCCGCGCCGCCGgccgcgc	6	1.2
ZF (SOX2-r131)	gaAAGGGGGCGGGGcCTCCCGcgccgcc	6	1.2
ZF (SOX2-r123)	ttTGCATGAAAGGGGGCGGGgcctcccg	6	1.2
ZF (SOX2-98)	cgGCAGCGAGGCTGGGCTCGagtggagg	6	2.6
ZF (SOX2-92)	cgAGGCTGGGCTCGAGTGGAggagccgc	6	1.6
**ZF (SOX2-88)**	**gcTGGGCTCGAGTGGAGGAGccgccgcg**	**6**	**2.3**
ZF (SOX2-84)	ggCTCGAGtGGAGGAGCCgccgcgcgct	5	1.9
ZF (SOX2-76)	tgGAGGAGCCGCCGCGCGCTgattggtc	6	1.5
ZF (SOX2-64)	cgCGCGCTGATTGGTCGctagaaaccca	5	1.2
ZF (SOX2-58)	ctGATTGGTCGCTAGAAacccatttatt	5	1.4
ZF (SOX2-9)	ggATGGTTGTCtATTAACttgttcaaaa	5	1.3
ZF (SOX2-r109)	cgCTGCCGGGTTTTGCAtgaaaggggc	5	1.6
**ZF (SOX2-r51)**	**atAAATGGGTTtCTAGCGaccaatcagc**	**5**	**2.0**
ZF (SOX2-r40)	ggGGGCTGtCAGGGAaTAAATGggtttc	6	1.7
ZF (SOX2-r31)	atGTGACGGGGGCTGTCAGGgaataaat	6	1.5
ZF (SOX2+27)	caGGAGTTGTCAAGGCAGAGaagagagt	6	1.3

ZF (SOX2+37)	caAGGCAGaGAAGAGAGTGTTtgcaaaa	6	1.2
ZF (SOX2+48)	agAGAGTGTTTGCAAAAGGGggaaagta	6	1.6
ZF (SOX2+58)	tgCAAAAGGGGGAAaGTAGTTtgctgcc	6	1.2
ZF (SOX2+64)	agGGGGAAaGTAGTTtGCTGCCtcttta	6	1.9
ZF (SOX2+88)	ttTAAGACTAGGACTGAGAGaaagaaga	6	1.4
ZF (SOX2+92)	agACTAGGACTGAGAGAAAGaagaggag	6	1.6
ZF (SOX2-r3)	ttTTTGAACAAGTTaATAGACaaccatc	6	1.3
ZF (SOX2+132)	ggAGAGAAgTTTGAGCCCCAGgcttaag	6	1.2
ZF (SOX2+186)	caTCGGCGGCGGCAGGAnnnnnnnnnnn	5	1.3
ZF (SOX2+r144)	gaAAGGCTTAAGCCTGGGGCtcaaactt	6	1.9
ZF (SOX2+r150)	ttTTTGGAAAGGCTTAAGCCtggggctc	6	1.1
ZF (SOX2+r178)	cgCCGCCGATGATTGTTattattatttt	5	1.3

Supplementary Table 3. ZF-TFs for KLF4

Name	Target Site	Fingers	KLF4 activation (Fold)
ZF (KLF4-698)	ttGAGGCTcCCAGTTCACGCTgcacagt	6	1.2
ZF (KLF4-698)	tcCCAGTTCACGCTGCACAGtgctgggc	6	1.6
ZF (KLF4-627)	ctCAGCAGaAGGGCCCGCGAGgaaccgt	6	1.3
ZF (KLF4-620)	gaAGGGCCCGCGAGGAACCGtgcgaggt	6	1.5
ZF (KLF4-614)	ccCGCGAGGAACCGTGCGAGgtcaggcc	6	1.5
ZF (KLF4-608)	agGAACCGTGCGAGGTCAGGccgagcgc	6	1.3
ZF (KLF4-574)	cgCCACCGGCACTGGCGCTGagcgacga	6	2.0
ZF (KLF4-568)	cgGCACTGGCGCTGAGCGACgagagcgg	6	2.6
ZF (KLF4-556)	tgAGCGACGAGAGCGGActcctgcgagc	5	1.1
ZF (KLF4-547)	agAGCGGACTCCTGCGAGCGcagagagg	6	1.2
ZF (KLF4-507)	gaGGGCTGGATGAGtCACGCGgataatc	6	1.1
ZF (KLF4-455)	gcGCGGCAAGCGCGTATGCTagcagggg	6	1.2
ZF (KLF4-449)	caAGCGCGTATGCTAGCAGGggtgcgga	6	1.3
ZF (KLF4-446)	gcGCGTATGCTAGCAGGggtgcggacgc	5	1.0
ZF (KLF4-443)	cgTATGCTAGCAGGGGTGCGgacgcgtg	6	1.1
ZF (KLF4-437)	ctAGCAGGGGTGCGGACGCGtgaccgtg	6	1.8
ZF (KLF4-380)	gcTTCGAACCCAGGGAGCCGacaatggc	6	1.2
ZF (KLF4-375)	gaACCCAGGGAGCCgACAATGgcggtga	6	1.6
ZF (KLF4-375)	ccCAGGGAGCCGACAATGGCggtgagta	6	1.4
ZF (KLF4-368)	ggGAGCCGACAATGGCGGTGagtacggc	6	1.1
ZF (KLF4-366)	gaGCCGACAATGGCGGTGAGtacggccc	6	0.9
ZF (KLF4-362)	cgACAATGGCGGTGAGTACGgccctggt	6	1.5
ZF (KLF4-136)	taGCTGCCATAGCAACGATGgaagggag	6	0.9
ZF (KLF4-130)	ccATAGCAACGATGGAAGGGagcctcgg	6	1.1
ZF (KLF4-122)	acGATGGAAGGGAGcCTCGGGgggggcg	6	2.9
ZF (KLF4-114)	agGGAGCCTCGGGGGGGGCGgagagaag	6	1.1
ZF (KLF4-109)	gcCTCGGGGGGGGCGGAGAGaagaaagg	6	1.0
ZF (KLF4-49)	agGCTGTAGCGAAGGAAGTTataagtaa	6	1.3
ZF (KLF4-16)	gcGCGCCGGCGGCCgGCAGTTtcccgac	6	1.6
ZF (KLF4-3)	cgGCAGTTTCCCGACCAGAGagaacgaa	6	1.0
ZF(KLF4+17)	agAGAACGAACGTGTCTGCGggcgcgcg	6	1.1
ZF (KLF4+23	cgAACGTGTCTGCGGGCGCGcggggagc	6	1.7
ZF (KLF4+29)	tgTCTGCGGGCGCGCGGGGAgcagaggc	6	2.3
ZF (KLF4+76)	acCGGGAGCCGCCGAGTGACcctccccc	6	1.5
**ZF (KLF4+134)**	**ccGTGGCCcGCGCCCATGGCCgcgcgcg**	**6**	**6.5**
ZF (KLF4-r750)	caGGCGGAAAGAGGCACGAAtggggagt	6	1.5
**ZF (KLF4-r739)**	**ttCCGCCGgGAACAGGCGGAAagaggca**	**6**	**3.1**
ZF (KLF4-r716)	nnAGAAGGCCAGAGGAGtgttccgccgg	5	0.8
ZF (KLF4-r696)	gtGCAGCGtGAACTGGGAGCCtcaagag	6	1.4
ZF (KLF4-r666)	nnGGAGAGGAGGCAGTGcccagcactgt	5	1.3
ZF (KLF4-r587)	ccGGTGGCGGTGCCGGCGCTcggcctga	6	1.6
ZF (KLF4-r576)	caGCGCCAGTGCCGGTGGCGgtgccggc	6	1.1
ZF (KLF4-r545)	tcTGCGCTCGCAGGAGTCCGctctcgtc	6	1.1
ZF (KLF4-r543)	tcTCTGCGCTCGCAGGAGTCcgctctcg	6	1.1
ZF (KLF4-r482)	gcGCGCTGGAGAAGAGCGCGattatccg	6	2.7
ZF (KLF4-r447)	caCCCCTGCTAGCATACGCGcttgccgc	6	0.8
ZF (KLF4-r423)	gcGGGCGGCACGGTCACGCGtccgcacc	6	1.2
ZF (KLF4-r412)	ctGGTGGGgTCAGCGGGCGGCacggtca	6	1.6
ZF (KLF4-r406)	gaAGACTGGTGGGGTCAGCGggcggcac	6	0.8
ZF (KLF4-r344)	cgGTCGCTgCGCGACCAGGGCcgtactc	6	0.8
ZF (KLF4-r326)	ggAACCGGGCGCAGGTTCGGtcgctgcg	6	1.1
ZF (KLF4-r320)	ncGCGAGGAACCGGGCGCAGgttcggtc	6	1.2

ZF (KLF4-r319)	nnCGCGAGGAACCGGGCGCAggttcggt	6	1.5
ZF (KLF4-r21)	ngCCGGCCgCCGGCGCGCGTTccttact	6	1.8
ZF (KLF4+r80)	ggGAGGGTCACTCGGCGGCTcccggtgc	6	0.5
ZF (KLF4+r127)	tgGGCGCGgGCCACGGGCGGGtgggagg	6	1.6
ZF (KLF4+r131)	ccATGGGCGCGGGCCACGGGcgggtggg	6	1.6
ZF (KLF4+r133)	nnCCATGGGCGCGGGCCACGggcgggtg	6	1.2
ZF (KLF4+r156)	ggTGAGTTGTGTGGAGCGCGcgcggcca	6	1.0
ZF (KLF4+r162)	ggACTCCGgTGAGTTGTGTGGagcgcgc	6	1.6
ZF (KLF4+r175)	ggCGCAAGGCGCGGACTCCGgtgagttg	6	2.3

Supplementary Table 4. ZF-TFs for MYC

Name	Target Site	Fingers	c-myc activation (Fold)
ZF (c-myc-797)	ccCGCACGGGGCCCCACGGAagcctgag	6	0.9
ZF (c-myc-784)	ccACGGAAgCCTGAGCAGGCGgggcagg	6	1.0
ZF (c-myc-765)	cgGGGCAGGAGGGGcGGTATCtgctgct	6	1.1
ZF (c-myc-758)	ggAGGGGCGGTATCtGCTGCTttggcag	6	1.1
ZF (c-myc-752)	gcGGTATCtGCTGCTTTGGCAgcaaatt	6	1.2
ZF (c-myc-729)	aaATTGGGgGACTCAGTCTGGgtggaag	6	0.9
ZF (c-myc-709)	ggTGGAAGgTATCCAATCCAGatagctg	6	0.9
ZF (c-myc-694)	atCCAGATaGCTGTGCATACAtaatgca	6	0.9
ZF (c-myc-604)	acGTGGCAaTGCGTTGCTGGGttatttt	6	1.0
ZF (c-myc-409)	caGCCTGGTACGCGcGTGGCGtggcggt	6	1.1
ZF (c-myc-403)	ggTACGCGcGTGGCGTGGCGGtgggcgc	6	0.9
ZF (c-myc-323)	tcACAGGACAAGGAtGCGGTTtgtcaaa	6	0.9
ZF (c-myc-295)	caGTACTGcTACGGAGGAGCAgcagaga	6	1.1
ZF (c-myc-244)	caAAAGAAaATGGTAGGCGCGcgtagtt	6	1.3
ZF (c-myc-237)	aaATGGTAGGCGCGcGTAGTTaattcat	6	1.3
ZF (c-myc-184)	ctAGAGCTAGAGTGcTCGGCTgcccggc	6	1.4
ZF (c-myc-24)	aaTGCGAGGGTCTGGACGGCtgaggacc	6	1.9
ZF (c-myc-23)	atGCGAGGGTCTGGACGGCTgaggaccc	6	2.1
**ZF (c-myc-18)**	**agGGTCTGGACGGCTGAGGAcccccgag**	**6**	**2.4**
**ZF (c-myc-17)**	**ggGTCTGGACGGCTGAGGACccccgagc**	**6**	**2.6**
ZF (c-myc-4)	tgAGGACCCCCGAGcTGTGCTgctcgcg	6	2.1
ZF (c-myc+97)	ttGGCGGGAAAAAGAACGGAgggaggga	6	1.0
ZF (c-myc+110)	gaACGGAGGGAGGGATCGCGctgagtat	6	2.0
ZF (c-myc+112)	acGGAGGGAGGGATCGCGCTgagtataa	6	1.3
ZF (c-myc+118)	ggAGGGATCGCGCTGAGTATaaaagccg	6	0.8
ZF (c-myc+135)	taTAAAAGCCGGTTTTCGGGgctttatc	6	1.3
ZF (c-myc+138)	aaAAGCCGGTTTTCGGGgctttatctaa	5	0.9
ZF (c-myc+141)	agCCGGTTTTCGGGGCTttatctaactc	5	1.4
ZF (c-myc-r801)	cgTGGGGCCCCGTGCGGGAGnnnnnnnn	6	1.6
ZF (c-myc-r753)	tgCCAAAGCAGCAGATACCGcccctcct	6	0.7
ZF (c-myc-r663)	caTTTGTTGGGGGGaGTCATGtattatg	6	1.0
ZF (c-myc-r603)	taACCCAGcAACGCATTGCCAcgtatac	6	0.9
ZF (c-myc-r555)	ggAATGATaGAGGCATAAGGAggaaaac	6	0.8
ZF (c-myc-r549)	atAGGGAGgAATGATaGAGGCAtaagga	6	0.9
ZF (c-myc-r535)	gaTGTTAGtGTAGATAGGGAGgaatgat	6	1.1
ZF (c-myc-r529)	gcGTGGGATGTTAGtGTAGATagggagg	6	0.9
ZF (c-myc-r520)	nnGTTCAGaGCGTGGGATGTTagtgtag	6	1.3
ZF (c-myc-r517)	cgCGCGTTcAGAGCGTGGGATgttagtg	6	0.9
ZF (c-myc-r513)	tgGGCGCGcGTTCAGAGCGTGggatgtt	6	1.1
ZF (c-myc-r511)	taATGGGCgCGCGTTcAGAGCGtgggat	6	1.1
ZF (c-myc-r504)	atTAATGGGCGCGCGTTcagagcgtggg	5	1.6
ZF (c-myc-r498)	aaGGGTATTAATGGGCGcgcgttcagag	5	1.0
ZF (c-myc-r416)	nnTACCAGGCTGCAGGGcgcctcgctaa	5	1.0
ZF (c-myc-r216)	nnAGAGAGCCGCATGAAttaactacgcg	5	0.9
ZF (c-myc-r216)	agAGTAAGAGAGCCgCATGAAttaacta	6	1.4
ZF (c-myc-r194)	ctCTAGCTcTAGGATGTAAACagagtaa	6	1.1
ZF (c-myc-r186)	gcCGAGCACTCTAGCTCTAGgatgtaaa	6	1.2
ZF (c-myc-r170)	naGACTCAGCCGGGCAGCCGagcactct	6	1.3
ZF (c-myc-r124)	ggAGGGGCgCTTATGGGGAGGgtgggga	6	1.4
ZF (c-myc-r120)	ncGGGAGGGGCGCTTATGGGgagggtgg	6	1.5
ZF (c-myc-r120)	nnCGGGAGgGGCGCTTATGGGgagggtg	6	1.8
ZF (c-myc-r118)	aaCCCGGGAGGGGCgCTTATGgggaggg	6	1.0

ZF (c-myc-r59)	ggGCGGAGaTTAGCGAGAGAGgatcttt	6	1.0
ZF (c-myc-r58)	tgGGCGGAGATTAGCGAGAGaggatctt	6	1.0
ZF (c-myc-r44)	ttATAAAGGGCCGGTGGGCGgagattag	6	0.8

Supplementary Table 5. ZF-TFs for miR302/367

Name	target site	Fingers	miR302 activation (Fold)
ZF(miR302-r391)	agATTGCTTCCACGgGGTGTTttgttct	6	0.4
ZF(miR302-r349)	tgTTTGAAGAAGCAtcTACATGttaaat	6	1.0
ZF(miR302-347)	acAGAGTTcCTCCAGATAGAAacacaat	6	1.4
ZF(miR302-r310)	gaGCCGAGaAAGGCAtTGTGTTtctatc	6	1.5
ZF(miR302-r272)	tgATGGCAtaGATTAGGGTAACaatgag	6	0.5
**ZF(miR302-r270)**	**ttTGATGGcATAGATtAGGGTAacaatg**	**6**	**2.2**
ZF(miR302-224)	ttTAAGAGGAAGATatCTTGTGgtaatg	6	1.3
**ZF(miR302-218)**	**agGAAGATatCTTGTGGTAATGgtttta**	**6**	**8.7**
ZF(miR302-209)	tcTTGTGGtaATGGTTTTAGCTgttaac	6	1.0
ZF(miR302-r175)	caGATGTCAATGTTaACAGCTaaaacca	6	0.9
ZF(miR302-153)	aaAAAGGAtCCAGACccACCCAGgatca	6	2.1
ZF(miR302+8)	nnTCTGGAGGAGAACACGAAtctttggg	6	2.1
ZF(miR302+r11)	tcCAGAAGggTAAAAGGCAGGGacttca	6	3.5
ZF(mir302+r14)	tcCTCCAGaAGGGTAaAAGGCAggact	6	2.3
ZF(miR302+r108)	atTTAGAGcTGAGGAgAAAGAAaacaaa	6	1.0
ZF(miR302+129)	aaGTTGTATGTTGGGTGGGCtcccttca	6	1.2
ZF(miR302+162)	aaCATGGAAGTGCTtTCTGTGactttaa	6	0.6
ZF(miR302+r167)	ctTCCATGttAAAGTTGAAGGGagccca	6	0.8
ZF(miR302+201)	tcCATGTTtTAGTAGgAGTGAAtccaat	6	1.2
ZF(miR302+267)	ggGATCCCcTTTGCTttAACATGggggt	6	0.8
ZF(miR302+274)	ccTTTGCTttAACATGGGGGTAcctgct	6	1.2
ZF(miR302+279)	gcTTTAACATGGGGgTACCTGctgtgtg	6	0.8
ZF(miR302+282)	ttAACATGGGGGTAcCTGCTGtgtgaaa	6	3.4
ZF(miR302+r359)	aaGGTGTGCTGGCTtGGAGACacctcca	6	0.9
ZF(miR302+r447)	gaGCCCAGtCTTGGAaAAAGTTagaatc	6	0.4
ZF(miR302+r454)	tgGTGGGGaGCCCAGtCTTGGAaaaagt	6	1.4
ZF(miR302+459)	ntTAAACGTGGATGTACTTGctttgaaa	6	0.7
ZF(miR302+r468)	caTCCACGTTTAAGTGGTGGggagccca	6	1.4
ZF(miR302+r488)	ctTTAGTTtcAAAGCAAGTACAtccacg	6	1.2
ZF(miR302+488)	taAAGAAGTaAGTGCTTCCATGttttgg	6	0.4
ZF(miR302+496)	taAGTGCTTcCATGTTTTGGTGatggta	6	0.7
ZF(miR302+644)	acATGGAGgCACTTGcTGTGACatgaca	6	1.6
ZF(miR302+647)	tgGAGGCAcTTGCTGtGACATGacaaaa	6	1.2
ZF(miR302+r732)	tgGGCGTTAACGCAATTGCTgattaggt	6	0.5
ZF(miR302+r734)	tgTGGGCGttAACGCAATTGCTgattag	6	0.7
ZF(miR302+736)	acACTGTGtGCAGTTcTTGGCTacaggc	6	0.7
ZF(miR302+r738)	acAGTGTGGGCGTTAACGCAattgctga	6	0.9
ZF(miR302+r740)	acACAGTGtGGCGTTAACGCAattgct	6	0.3
ZF(miR302+r742)	gcACACAGtGTGGGCGTTAACgcaattg	6	1.5
ZF(miR302+743)	gtGCAGTTcTTGGCTaCAGGCCattact	6	0.5
ZF(miR302+750)	tcTTGGCTaCAGGCCaTTACTGttgcta	6	0.6
ZF(miR302+r765)	gtAATGGCCTGTAGcCAAGAActgcaca	6	1.4
ZF(miR302+819)	caATGGTGATGGATtGTTAAGccaatga	6	1.1
ZF(miR302+1026)	taAAAAAGcTCTCCGtTAAGACatgggg	6	1.3
ZF(miR302+1033)	gcTCTCCGtTAAGACATGGGGctgtgaa	6	1.0
ZF(miR302+1035)	tcTCCGTTaaGACATGGGGCTGtgaaat	6	0.3
ZF(miR302+r1180)	aaGCATCAGAAGCTtcCAAGGAaaatag	6	2.2
ZF(miR302+1218)	ctTTTGATCGGGGAGCTAGGgattctgc	6	0.8
ZF(miR302+1221)	ttGATCGGGGAGCTAGGGATtctgcatc	6	2.8
ZF(miR302+r1227)	tcCCCGATCAAAAGtAAAGAAaattatt	6	2.6

ZF(miR302+r1234)	ccCTAGCTcCCCGATCAAAAGtaaagaa	6	0.6
ZF(miR302+r1290)	ctTACCTGaAGTCAGAACAGGgaagagg	6	1.3
ZF(miR302+r1309)	acAGAGTTGAAGCAtCTAGCTtacctga	6	4.4
ZF(miR302+r1316)	aaACGGGAcAGAGTTGAAGCAtctagct	6	1.3
ZF(miR302+r1317)	aaAACGGGacAGAGTTGAAGCAtctagc	6	0.7
ZF(miR302+1342)	ctTTTGGGgtTCAGTGGACTCAatgtca	6	1.3
ZF(miR302+1343)	ttTTGGGGtTCAGTGGACTCAatgtcag	6	2.4
ZF(miR302+1353)	caGTGGACtcAATGTCAGAGCCctcaga	6	1.5
ZF(miR302+r1473)	agGCCGAAtgAAAAAGGAAGACaatatc	6	1.1
ZF(miR302+r1479)	gaAGAAAGGCCGAAtgAAAAAGgaagac	6	1.3

Supplementary Table 6. qPCR primers for ChIP

Primer pair*	Forward	Reverse
MYT	ACTCCACCGGACTGGGACACC	GCTACCCATGAACCCTTGGGGC
P-2334	CTGTGTTCACTTCTCGGCCT	TTCCGAAGCATGTTCCTCCC
P-1748	GCAGAGGAGGTGGAGAGTGA	GCACTAGACCAGCAGCATGA
P-1362	AATGGCCTTGGCTGGACAAT	AGGCCCATTCAAGGGTTGAG
P-1175	CTTGCTCCCCTCTCAACCAA	TAGGGCTGGAGGCCTGG
P-834	TCCCCCGATCCATCCAGAAT	GGCCTTGTCTGGCAGTCTAC
P+43	AAGGCGAAATCCGAAGCCA	CTTCGCAAGCCCTCATTTCAC
P+261	ACTAGCCCCACTCCAACCTG	GCTCTGAGGTGTGGGGGATT

* OCT4 primer pairs are named for the midpoint of their amplicon. For example, P-2334 amplifies the sequence surrounding position -2334 relative to the TSS.

Supplementary Figure Legends

Supplementary Figure 1. Results for engineered ZF-TFs targeting endogenous SOX2, KLF4, c-MYC, and miR302/367 in HEK293 cells. Procedure and designations are the same as in Figure 1. (A) SOX2, (B) KLF4, (C) c-MYC, (D) miR302/367.

Supplementary Figure 2. ChIP controls. (A) Western analysis was performed with protein extracts from 12-well cultures transfected in parallel with those used for FLAG-ChIP using the same anti-FLAG M2 antibody, as described in Materials and Methods. Asterisk marks ZF-TF band. (B) Positive control ChIP. Procedure was as in Figure 1B. One-third of each sheared chromatin preparation was immunoprecipitated with anti-H3K27me3 and target DNA detected by qPCR with the same primers as used for FLAG-ChIP-qPCR.

Supplementary Figure 3. Expression of ZF-TFs with alternative activation domains. Western analysis with anti-FLAG M2 antibody and protein extracts from HEK293 cells transfected with each of the ZF-TF constructs used in Figure 3, processed as described in Materials and Methods. Asterisks mark ZF-TF bands. Note that VP64 is a tandem repeat with 4 copies of the VP16 minimal activation domain, making VP64 is a shorter peptide than VP16.

Supplementary Figure 4. DNase hypersensitive regions in various cell lines. DNase hypersensitivity data for OCT4 (A), SOX2 (B), KLF4 (C), c-MYC (D) and miR302/367 (E) from two embryonic stem cells and several somatic cell lines was downloaded from the UCSC Genome Browser (32,35).

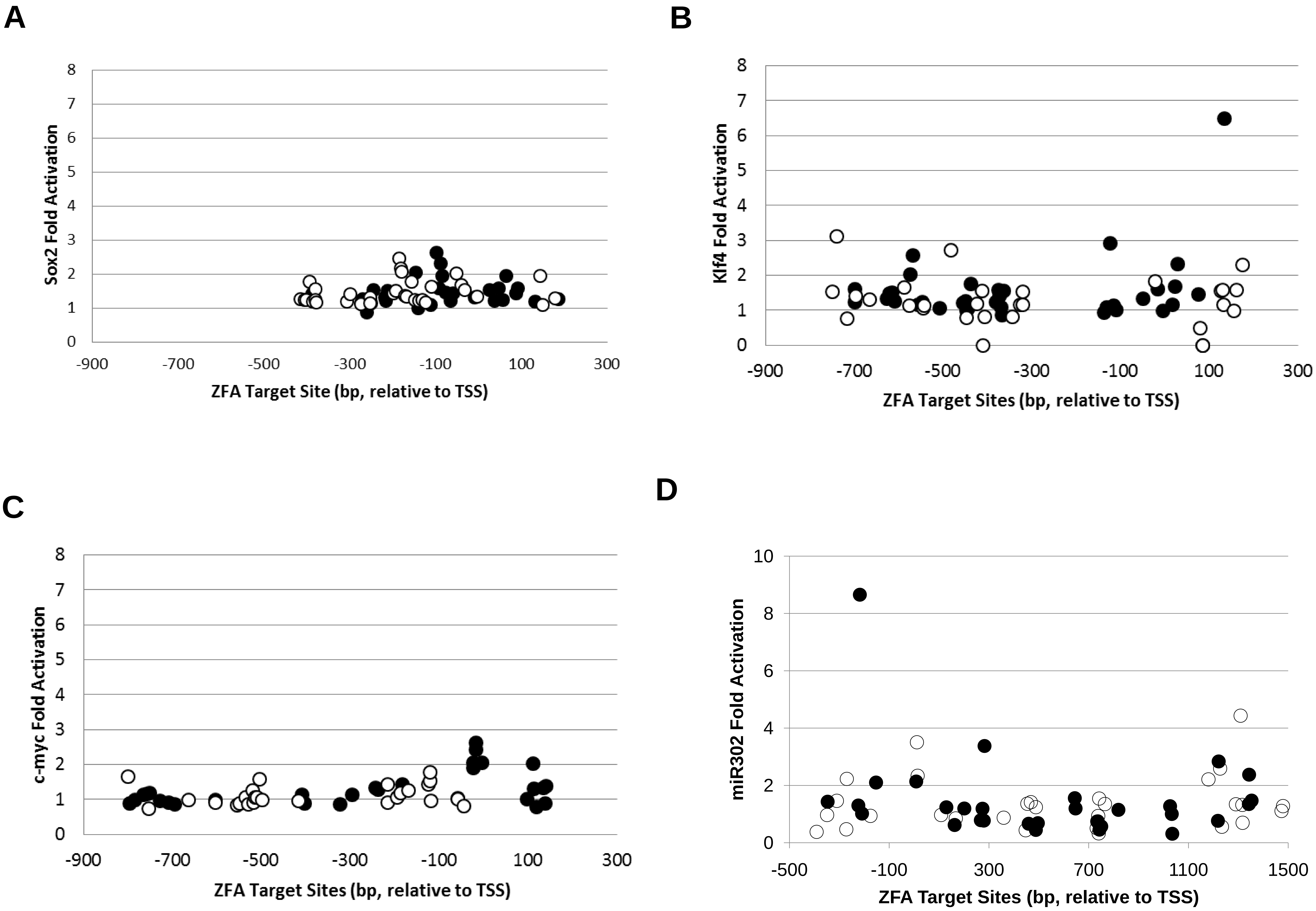

Supplementary Figure 1

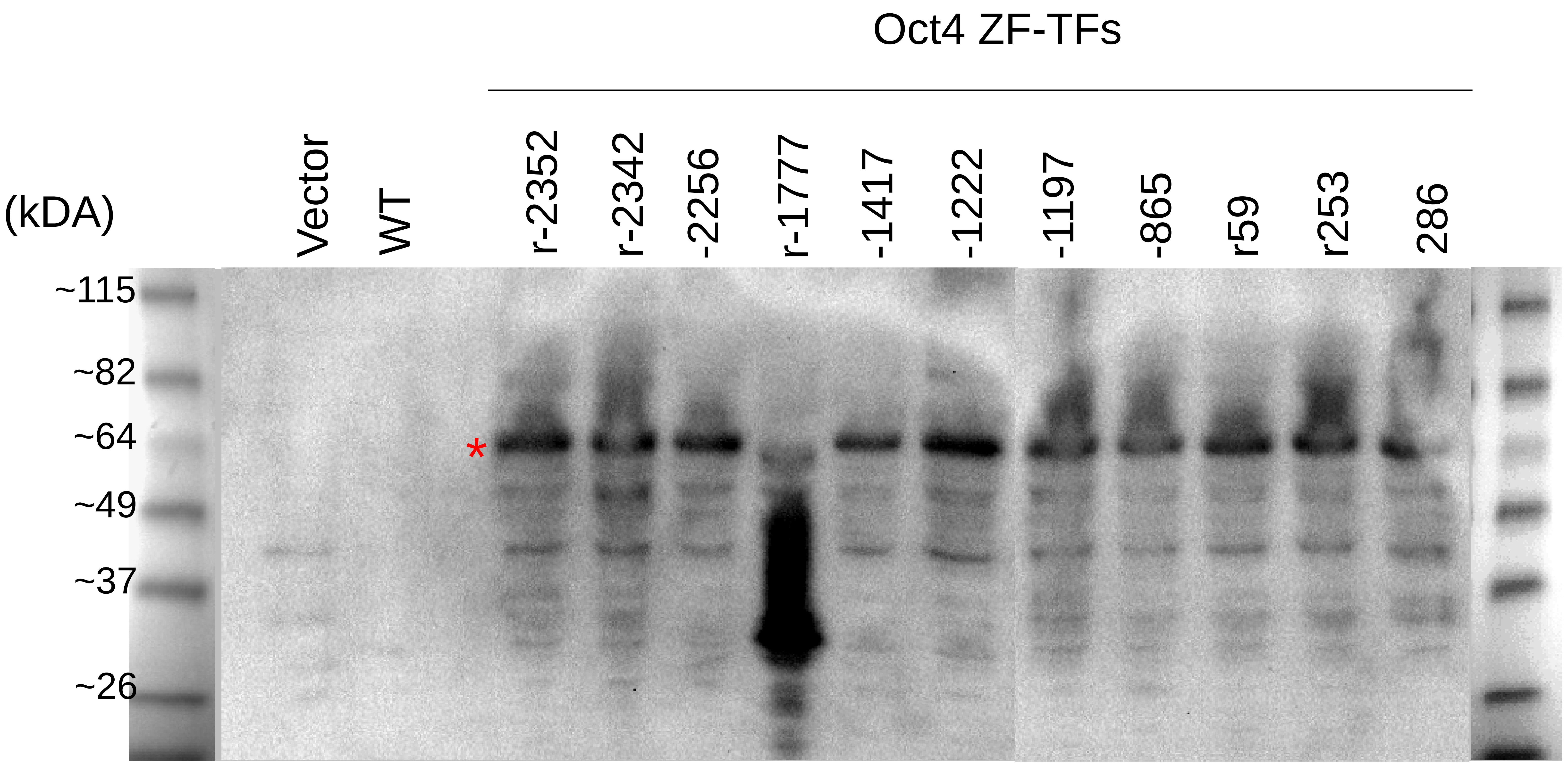

Supplementary Figure 2A

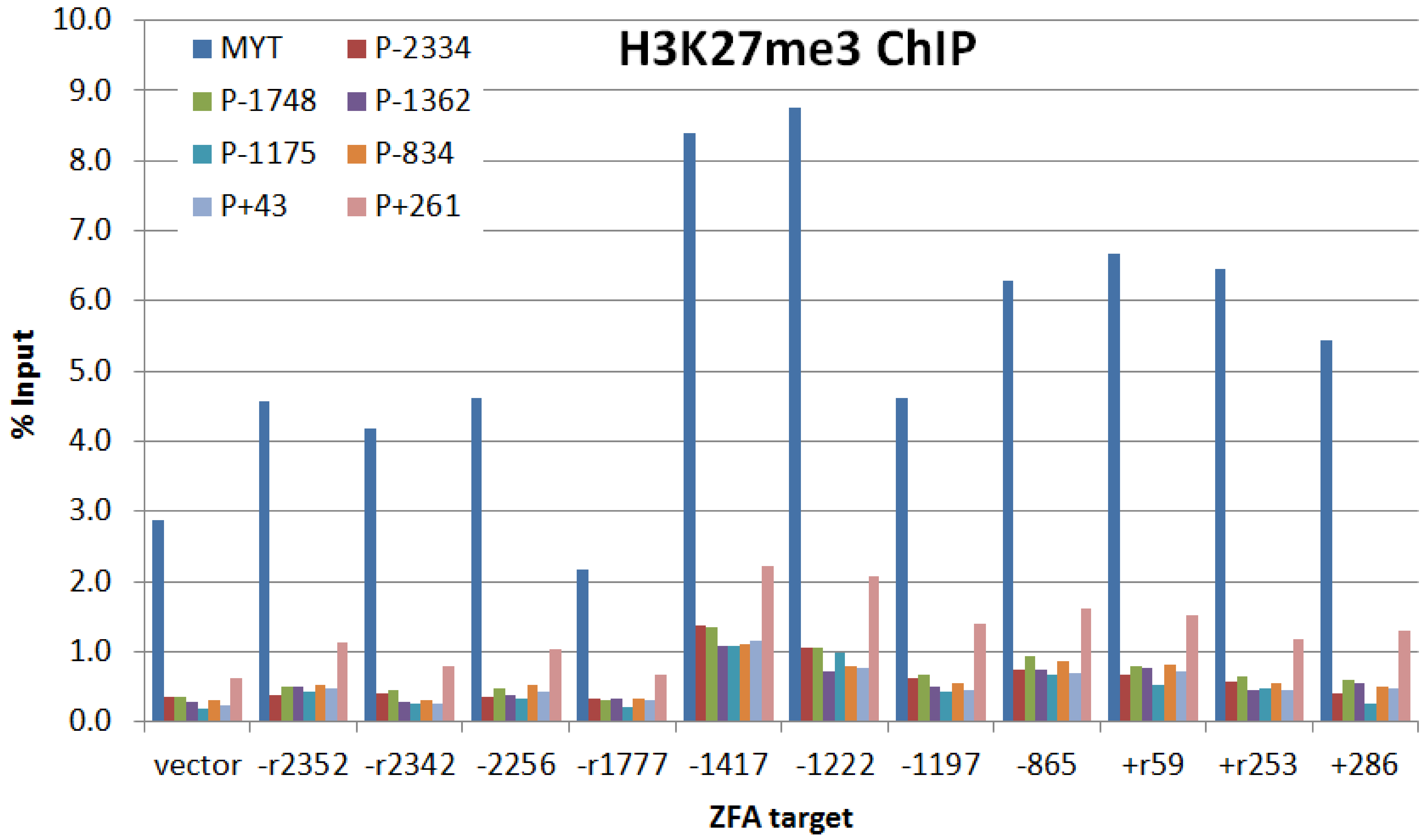

Supplementary Figure 2B

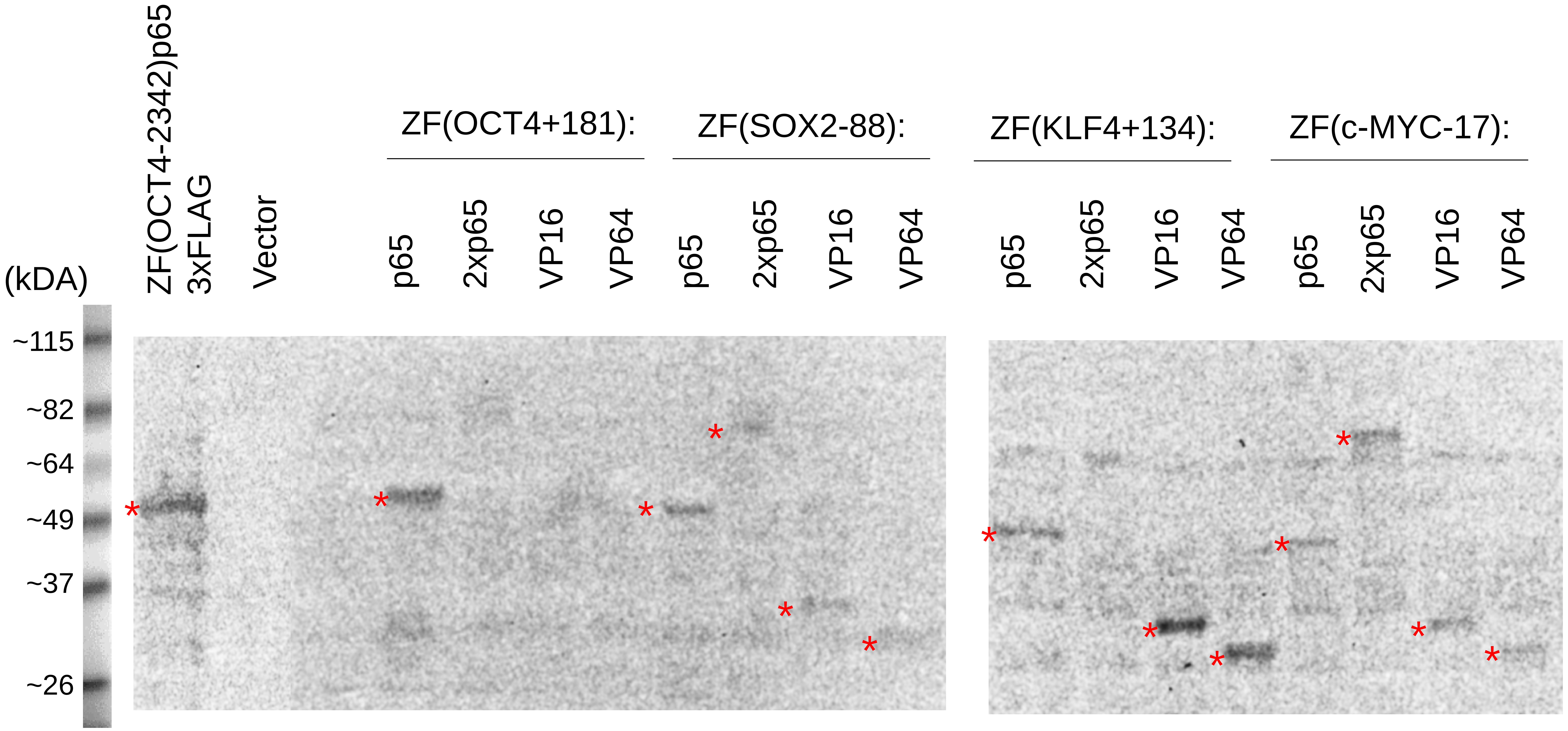

Supplementary Figure 3

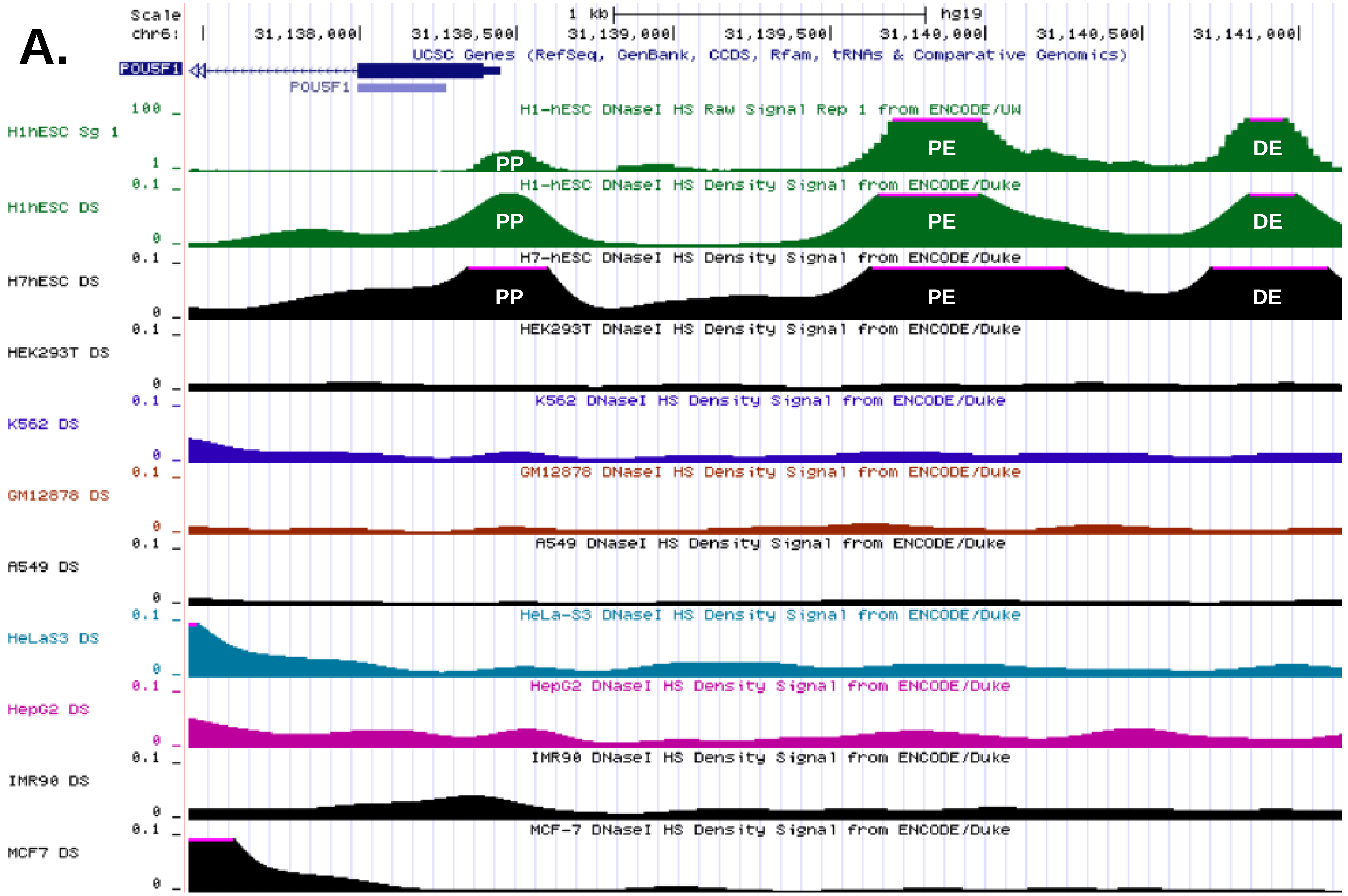

Supplementary Figure 4A

B.

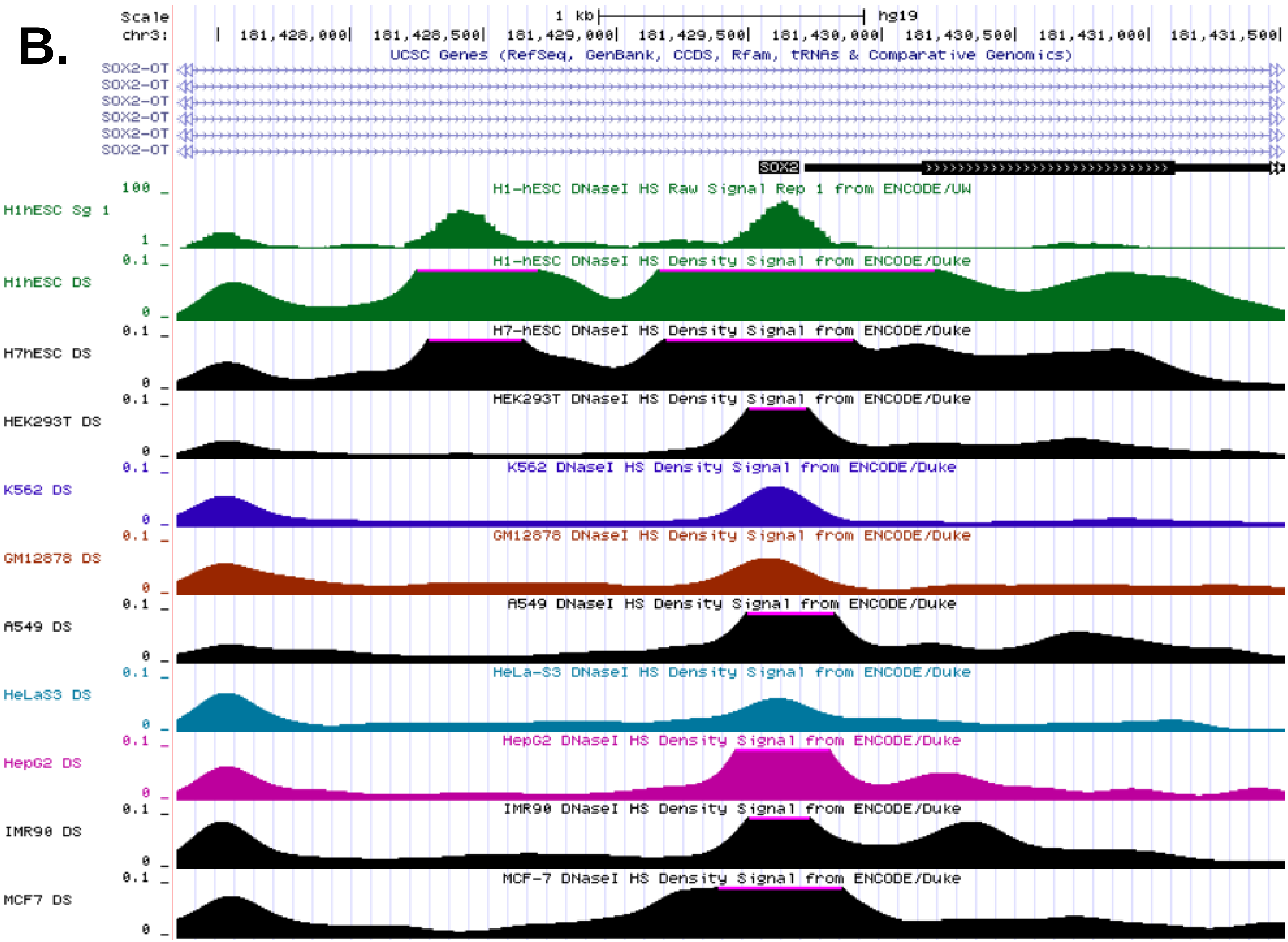

Supplementary Figure 4B

C.

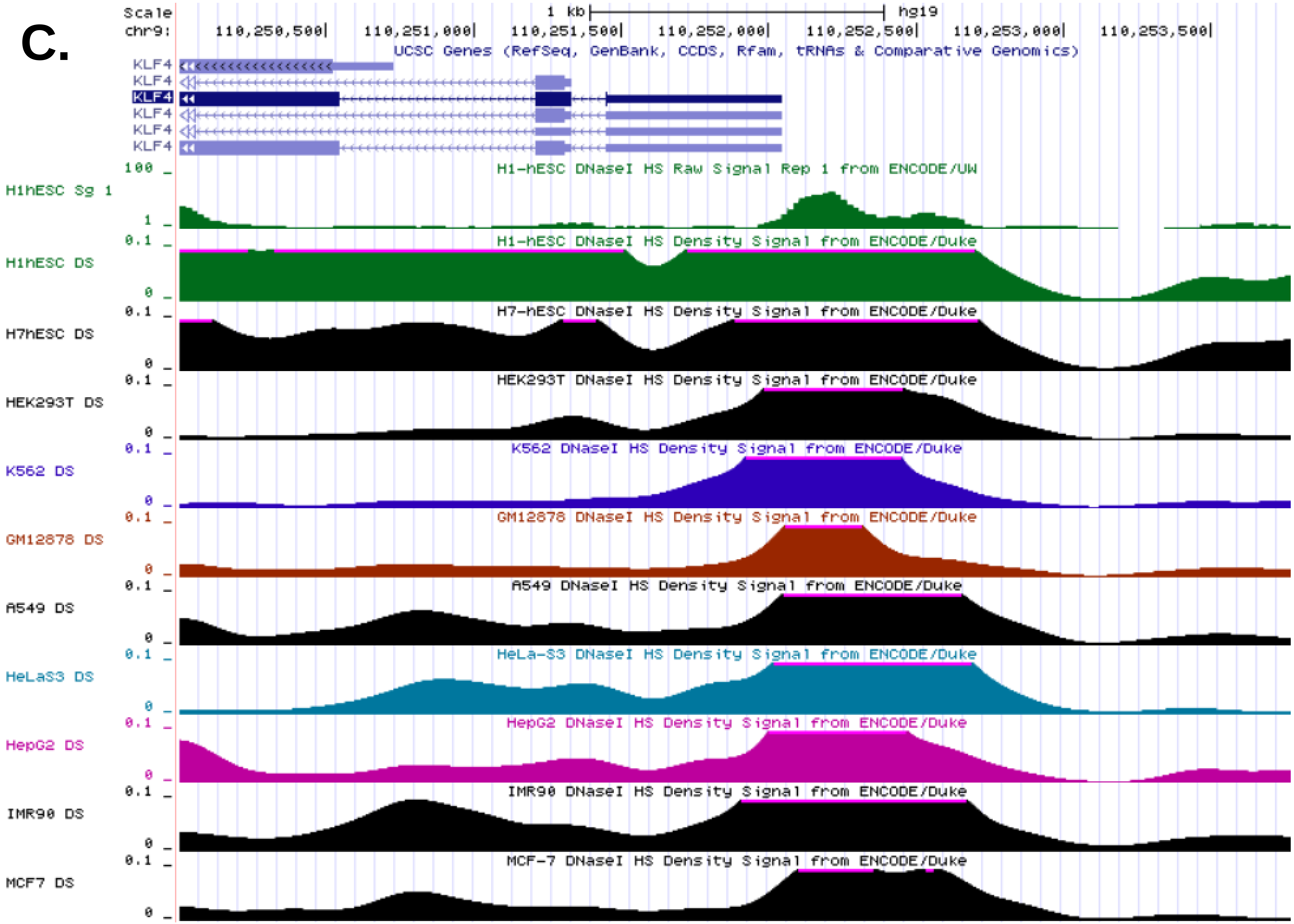

Supplementary Figure 4C

D.

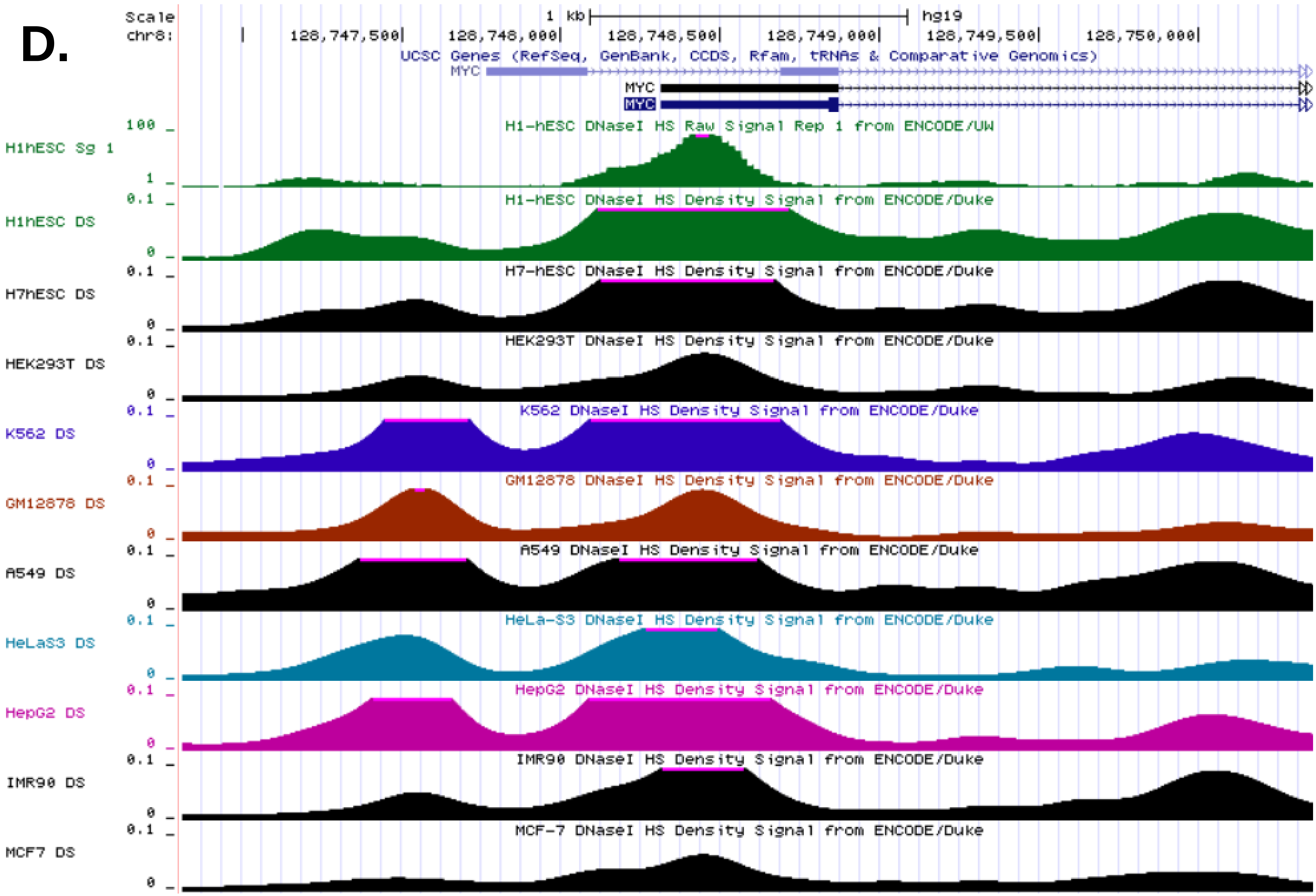

Supplementary Figure 4D

E.

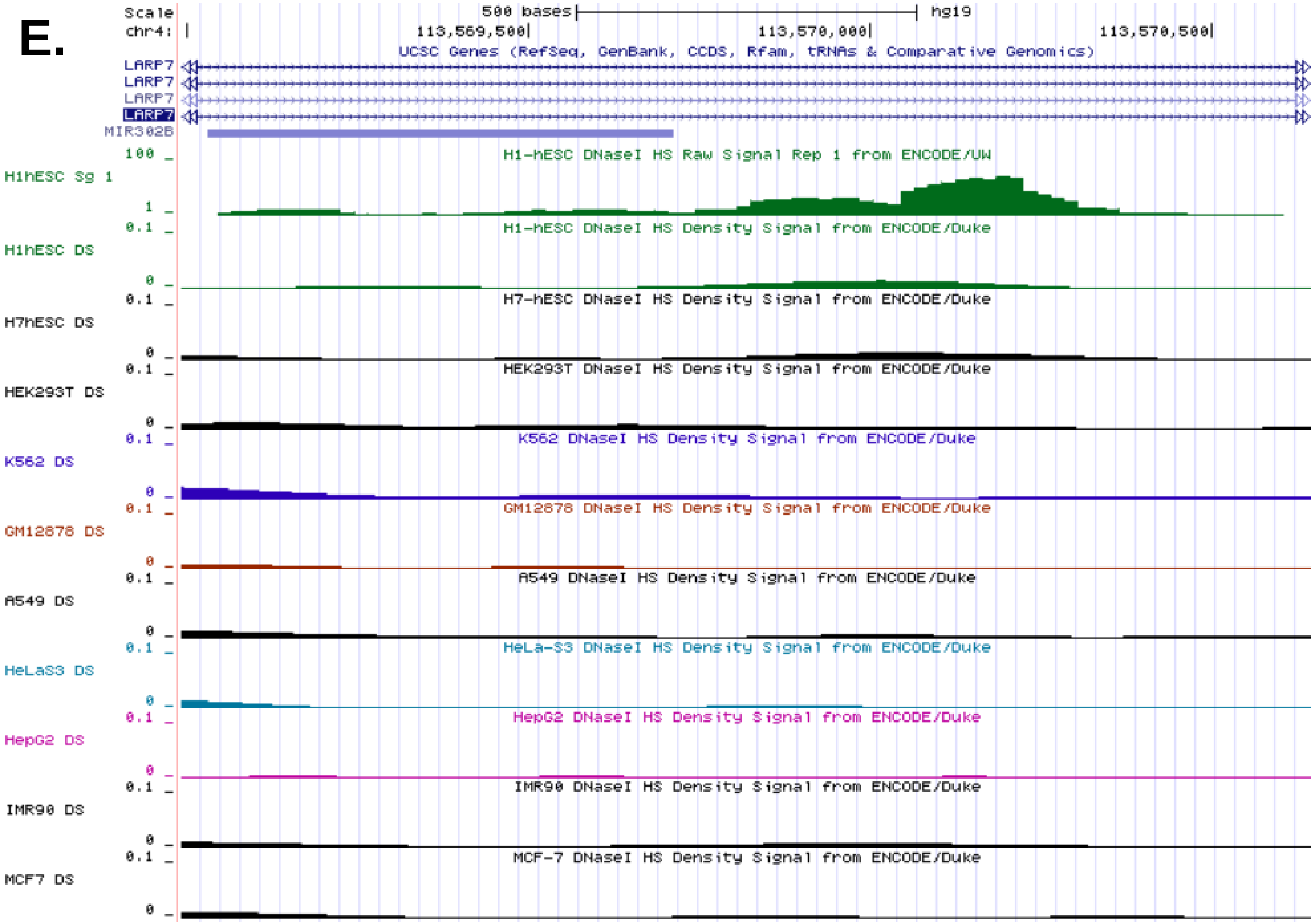

Supplementary Figure 4E
